# Reduced mortality and morbidity associated with metformin and SGLT2 inhibitor therapy in patients with type 2 diabetes mellitus and cirrhosis

**DOI:** 10.1186/s12876-023-03085-8

**Published:** 2023-12-19

**Authors:** Daniel J Huynh, Benjamin D Renelus, Daniel S Jamorabo

**Affiliations:** 1 Renaissance School of Medicine at Stony Brook University, Stony Brook, NY USA; 2 Division of Gastroenterology and Hepatology, Morehouse School of Medicine, Atlanta, GA USA; 3 Division of Gastroenterology and Hepatology, Stony Brook Medicine, 101 Nicolls Road, Health Sciences Center, Stony Brook, NY 11794-8167 USA

**Keywords:** Cirrhosis, Diabetes mellitus, Metformin, Sodium-glucose co-transporter-2 inhibitors, Mortality, Hepatic decompensation, Hepatocellular Carcinoma

## Abstract

**Introduction:**

Evidence for dual antidiabetic therapy in type 2 diabetes mellitus patients with cirrhosis is limited. This study compared 5-year mortality, composite hepatic decompensation risk, and hepatocellular carcinoma occurrence in patients with diabetes and cirrhosis who were either on metformin monotherapy or on dual metformin and sodium-glucose co-transporter-2 inhibitor (SGLT2-I) therapy.

**Methods:**

This retrospective study used the TriNetX Research Network to identify propensity score-matched patients treated with either metformin or dual metformin and SGLT2-I therapy. Our outcomes were all-cause mortality, a composite of hepatic decompensation events, and hepatocellular carcinoma (HCC) occurrence over 5 years. We estimated hazard ratios within each cohort with 95% confidence intervals (CI) and Kaplan-Meier estimates for time-to-event distributions with Log-rank tests. We were able to stratify our cohorts by age, sex, race, and ethnicity. We further investigated a subset of diabetic patients with cirrhosis due to MASH.

**Results:**

In our propensity score-matched cohorts of type 2 diabetes patients with cirrhosis, those on dual metformin and SGLT2-I therapy had decreased risk for mortality (HR 0.57, 95%CI 0.41–0.81), reduced composite risk of becoming decompensated (HR 0.63, 95%CI 0.43–0.93) and less than half the risk for developing HCC (HR 0.43, 95%CI 0.21–0.88) compared to those on mono metformin therapy. We did not find a difference between mono or dual therapy treatment for mortality, decompensation, or HCC risks in the subset of patients with MASH cirrhosis.

**Conclusion:**

Dual metformin and SGLT2-I treatment in type 2 diabetes patients with cirrhosis are associated with improved mortality and hepatic complications.

**Supplementary Information:**

The online version contains supplementary material available at 10.1186/s12876-023-03085-8.

## Introduction

Metformin is a standard initial treatment for type 2 diabetes mellitus (T2DM), though some patients require additional glucose-lowering agents due to disease progression [1]. Sodium glucose cotransporter-2 inhibitors (SGLT2-I) have been recommended as part of stepwise combination therapy for T2DM patients with established atherosclerotic cardiovascular disease (ASCVD), heart failure (HF), kidney disease, or those who are at risk for cardiorenal comorbidities [2]. Newer guidelines now recommend using SGLT2-I as part of a first-line glucose-lowering regimen in consideration of person-specific factors [1].

Cirrhosis is the third leading cause of death in adults aged 45–65 years [3]. It is a common comorbidity of T2DM, which is thought to be related to the progression of fatty liver and steatohepatitis [4]. Over time, patients with cirrhosis can develop decompensation, characterized by ascites, hepatic encephalopathy, and variceal bleeding, all of which in turn increase mortality risk [5, 6].

Moreover, other studies suggest that chronic hyperglycemia exacerbates liver disease and increases cirrhosis-related complications [7, 8]. Currently, there is scarce evidence for the utility of anti-diabetic medications in reducing morbidity and mortality in patients with T2DMand cirrhosis. Evidence for the use of SGLT2-I in patients with liver disease has been limited to metabolic dysfunction-associated steatotic liver disease (MASLD) and metabolic dysfunction-associated steatohepatitis (MASH) [9], previously called non-alcoholic fatty liver disease (NAFLD) and non-alcoholic steatohepatitis (NASH), respectively. Preclinical and clinical data showed that SGLT2-I treatment may improve liver histology and lab testing in MASH and MASLD patients [10, 11]. Furthermore, SGLT2-I promotes osmotic diuresis and targets neurohormonal pathways that benefit blood pressure [12]. There is reason to believe that SGLT2-I may also target pathways in patients with cirrhosis that slow fibrosis progression and reduce the risk for complications such as decompensation events [13] and hepatocellular carcinoma (HCC).

We sought to determine whether patients with cirrhosis and T2DM had a mortality benefit and reduced risk for complications such as decompensation and hepatocellular carcinoma when on dual therapy with metformin and an SGLT2-I versus on metformin alone.

## Methods

### Study population and design

We used the TriNetX database to build and analyze cohorts of patients with type 2 diabetes and cirrhosis on metformin monotherapy or metformin-SGLT2-I dual therapy. As such, we retrospectively analyzed de-identified patient data from fifty healthcare organizations (HCOs) globally, in particular demographic information, diagnostic and procedural information, and standard measurements such as labs, vital signs, and medications using standardized coding systems including International Classifications of Diseases, Tenth Revision [ICD-10] and Current Procedural Terminology [CPT] codes for diagnoses and procedures. We also used RxNorm codes for medication use and Logistical Observation Identifiers Names and Codes [LOINC] for lab values. Our study included patients treated from 1 March 2014 through 13 December 2022.

TriNetX LLC complies with section § 164.514(a) of the HIPAA Privacy Rule, contains only de-identified patient data, and has gained exemption from the Institutional Review Board approval. Our study used only de-identified patient records and did not involve collecting, using, or transmitting individually identifiable data. Further, TriNetX does not disclose data on participating HCOs to safeguard protected health information. More details of TriNetX networks have been previously described [14, 15].

### Data collection and outcomes

**Fig. 1:**
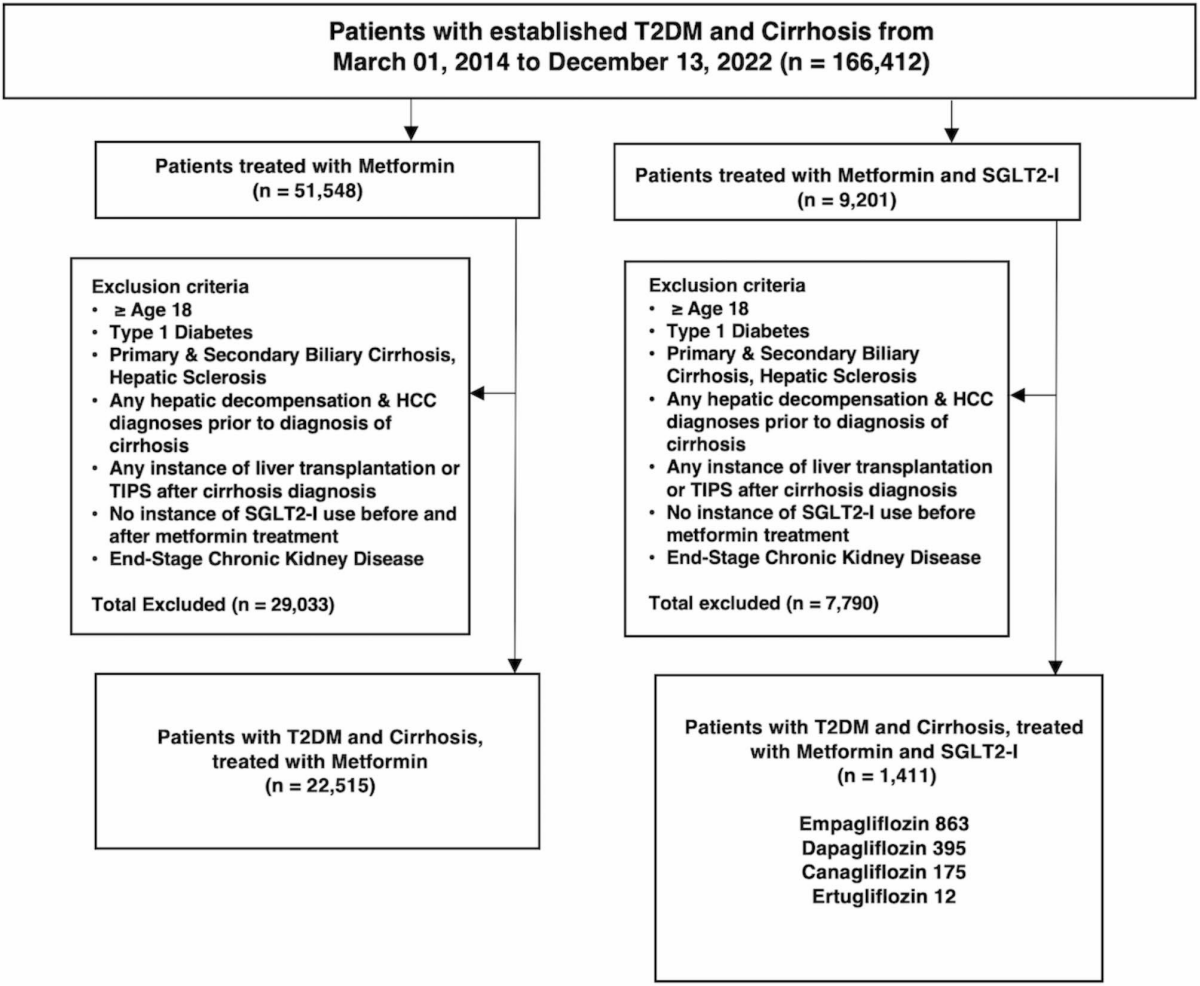
Cohort Creation for Metformin versus Metformin-SGLT2-I Groups; Cohort 1 included patients who were initiators of metformin without any instance of SGLT2-I in their electronic medical record (EMR). Cohort 2 inclusion included metformin initiators and additional SGLT2-I therapy. Both cohorts consisted of patients starting on March 01, 2014 to December 13, 2022. T2DM, Type 2 Diabetes Mellitus; SGLT2-I, Sodium glucose cotransporter-2 inhibitors; TIPS, Transvenous Intrahepatic Portosystemic Shunt; HCC, Hepatocellular Carcinoma

After confirming, we had patients who were at least the age of 18 with type 2 diabetes and cirrhosis from multiple etiologies (i.e., MASH, alcoholic, viral, and cryptogenic) and a subset of type 2 diabetes patients with MASH cirrhosis using ICD-10 codes. We excluded patients with primary and secondary biliary cirrhosis. After that, we identified a cohort who were on metformin monotherapy and another on metformin with SGLT2-I (i.e., canagliflozin, ertugliflozin, dapagliflozin, and empagliflozin) (Fig. 1). Patients were excluded if they initiated mono or dual therapy before either diabetes or cirrhosis diagnosis. We excluded patients with type 1 diabetes mellitus and those with a transhepatic intrajugular portosystemic shunt and liver transplantation after the diagnosis of cirrhosis. Chronic kidney disease is a contraindication to metformin initiation due to the risks of lactic acidosis; therefore, patients with stage 4 and 5 end-stage kidney disease were further excluded. We further excluded patients with decompensated cirrhosis at the chosen index with diagnosis codes for hepatic encephalopathy (HE), ascites, and variceal bleeding to define a cohort of patients with compensated cirrhosis at baseline. Patients with hepatic complications such as hepatocellular carcinoma (HCC) were also excluded if diagnosed before a cirrhosis diagnosis.

After exclusions, our cohort consisted of type 2 diabetes patients with compensated cirrhosis at baseline, of which 22,515 patients were in the monotherapy group, and 1,411 patients were in the dual therapy group (Fig. 1). We then undertook propensity-score matching (PS-matching) to build a monotherapy and a dual therapy cohort for direct comparison. We stratified patients further by sex, race, ethnicity, and age groups (Supplemental Table 1). In a separate analysis, we identified type 2 diabetes patients with cirrhosis due to MASH to similarly build mono- and dual-therapy cohorts for comparison (Supplemental Fig. 1). Liver disease severity was estimated with Fibrosis-4 (FIB-4) and Model for End-Stage Liver Disease (MELD-Na) scores. FIB-4 and MELD-Na scores for each respective cohort were calculated from baseline patient data before and after PS-matching (Supplemental Tables 2 and 3). Supplemental Table 4 fully describes study definitions, ICD-10 codes, and variables used to query patients in the TriNetX database.

Our primary outcome was mortality. Our secondary outcome was hepatic decompensation defined by a composite of ascites, HE, and esophageal or gastric variceal bleeding. Our tertiary outcome was the occurrence of HCC. All outcomes were recorded up to 5 years after the indexed monotherapy or dual therapy initiation.

### Statistical analyses

We identified covariates such as age, sex, race, ethnicity, labs, medications, surgical procedures, and comorbidities. We matched for drugs such as beta-blockers, calcium channel blockers, and hypoglycemics agents such as sulfonylureas, alpha-glucosidase inhibitors, dipeptidyl Peptidase-4 (DPP-4) inhibitors, thiazolidinediones, and glucagon-like peptide 1 receptor agonists (GLP1-RA) among others. Supplemental Tables 5 and 6 identify all covariates that were used for PS-matching. The date of lab values is concordant with the baseline characteristics. We then used the TriNetX platform to perform propensity score matching between the two cohorts, producing balanced monotherapy and dual therapy groups of 1,403 patients each (Table 1). The matching process was repeated for all type 2 diabetes patients with cirrhosis on monotherapy versus dual therapy by sex (women on metformin vs. women on metformin and SGLT2-I), race, ethnicity, and age groups. In our type 2 diabetes cohort of patients with cirrhosis due to MASH, patients on metformin and SGLT2-I were PS-matched to those on metformin only.

**Table 1:** Baseline Characteristics for T2DM Patients with Cirrhosis

	T2DM Cirrhosis					
**Baseline Characteristics**	Before Propensity Score Matching			After Propensity Score Matching		
	Metformin	Metformin + SGLT2-I	P	Metformin	Metformin + SGLT2-I	P
**Age (Mean ± SD)**						
Age at Index	58.56 ± 14.41	60.35 ± 11.67	< 0.001	60.55 ± 12.88	60.34 ± 11.66	0.642
**Sex**						
Men	10368 (46.05)	688 (48.76)	0.048	670 (47.75)	684 (48.75)	0.597
Women	11645 (51.72)	707 (50.11)	0.239	711 (50.68)	703 (50.11)	0.763
Unknown Gender	502 (2.23)	16 (1.13)	< 0.01	22 (1.57)	16 (1.14)	0.327
**Race**						
White	15267 (67.81)	985 (69.81)	0.118	1025 (73.06)	980 (69.85)	0.060
American Indian or Alaska Native	214 (0.95)	10 (0.71)	0.360	10 (0.71)	10 (0.71)	1.000
Asian	599 (2.66)	36 (2.55)	0.805	26 (1.85)	36 (2.57)	0.199
Black or African American	3278 (14.56)	195 (13.82)	0.444	177 (12.62)	194 (13.83)	0.343
Native Hawaiian or Other Pacific Islander	30 (0.13)	10 (0.71)	< 0.001	10 (0.71)	10 (0.71)	1.000
Unknown Race	3127 (13.89)	184 (13.04)	0.371	166 (11.83)	183 (13.04)	0.331
**Ethnicity**						
Hispanic or Latino	2570 (11.42)	176 (12.47)	0.226	170 (12.12)	174 (12.4)	0.818
Not Hispanic or Latino	14816 (65.81)	980 (69.45)	< 0.01	992 (70.71)	974 (69.42)	0.458
Unknown Ethnicity	5129 (22.78)	255 (18.07)	< 0.001	241 (17.18)	255 (18.18)	0.488
**Comorbidities**						
Acute kidney failure and chronic kidney disease	3813 (16.93)	437 (30.97)	< 0.001	427 (30.44)	431 (30.72)	0.870
Behavioral syndromes associated with physiological disturbances and physical factors	1194 (5.3)	111 (7.87)	< 0.001	101 (7.2)	111 (7.91)	0.475
Diseases of the digestive system	16811 (74.67)	1271 (90.08)	< 0.001	1247 (88.88)	1263 (90.02)	0.325
Endocrine, nutritional and metabolic diseases	19630 (87.19)	1334 (94.54)	< 0.001	1306 (93.09)	1326 (94.51)	0.117
Heart failure	2743 (12.18)	323 (22.89)	< 0.001	316 (22.52)	316 (22.52)	1.000
Hypertensive diseases	15006 (66.65)	1134 (80.37)	< 0.001	1086 (77.41)	1127 (80.33)	0.058
Ischemic heart diseases	4757 (21.13)	482 (34.16)	< 0.001	477 (34)	475 (33.86)	0.936
Mental and behavioral disorders due to psychoactive substance use	6860 (30.47)	472 (33.45)	0.018	446 (31.79)	469 (33.43)	0.354
Metabolic disorders	14180 (62.98)	1137 (80.58)	< 0.001	1131 (80.61)	1129 (80.47)	0.924
Other specified diabetes mellitus	948 (4.21)	84 (5.95)	< 0.01	81 (5.77)	83 (5.92)	0.872
Overweight, obesity and other hyperalimentation	9225 (40.97)	782 (55.42)	< 0.001	781 (55.67)	776 (55.31)	0.849
**Surgeries and Procedures**						
Surgery	15259 (67.77)	1122 (79.52)	< 0.001	1069 (76.19)	1114 (79.4)	0.041
Surgical Procedures on the Digestive System	5828 (25.89)	559 (39.62)	< 0.001	552 (39.34)	554 (39.49)	0.938
**Medications**						
Ace inhibitors	8683 (38.56)	706 (50.03)	< 0.001	688 (49.04)	701 (49.96)	0.624
Angiotensin ii inhibitor	4333 (19.25)	490 (34.73)	< 0.001	495 (35.28)	483 (34.43)	0.634
Antiarrhythmics	9014 (40.04)	780 (55.28)	< 0.001	766 (54.6)	772 (55.02)	0.820
Anticoagulants	7875 (34.98)	669 (47.41)	< 0.001	637 (45.4)	663 (47.26)	0.325
Antihypertensives,other	4042 (17.95)	360 (25.51)	< 0.001	354 (25.23)	356 (25.37)	0.931
Antihypoglycemics	6241 (27.72)	582 (41.25)	< 0.001	580 (41.34)	576 (41.06)	0.878
Antilipemic agents	10930 (48.55)	1007 (71.37)	< 0.001	996 (70.99)	1000 (71.28)	0.868
Beta blockers/related	9079 (40.32)	778 (55.14)	< 0.001	756 (53.89)	772 (55.02)	0.544
Calcium channel blockers	6017 (26.72)	508 (36)	< 0.001	486 (34.64)	504 (35.92)	0.477
Diuretics	9774 (43.41)	799 (56.63)	< 0.001	784 (55.88)	793 (56.52)	0.732
Platelet aggregation inhibitors	8412 (37.36)	767 (54.36)	< 0.001	757 (53.96)	760 (54.17)	0.910
Insulin	9395 (41.73)	864 (61.23)	< 0.001	847 (60.37)	857 (61.08)	0.699
a-carbose	54 (0.24)	10 (0.73)	< 0.001	9 (0.67)	7 (0.48)	0.413
Miglitol	9 (0.04)	0 (0)	0.319	7 (0.48)	0 (0)	< 0.01
Alogliptin	83 (0.37)	14 (1.02)	< 0.001	22 (1.06)	19 (0.92)	0.638
Linagliptin	529 (2.35)	67 (4.72)	< 0.001	90 (4.33)	89 (4.28)	0.939
Saxagliptin	157 (0.7)	17 (1.22)	< 0.01	33 (1.59)	23 (1.11)	0.178
Sitagliptin	2102 (9.34)	282 (20.1)	< 0.001	379 (18.25)	359 (17.28)	0.417
Nateglinide	34 (0.15)	6 (0.41)	< 0.01	7 (0.48)	7 (0.48)	1.000
Repaglinide	150 (0.67)	15 (1.06)	0.029	13 (0.92)	13 (0.96)	0.872
Glipizide	3062 (13.59)	331 (23.47)	< 0.001	305 (21.76)	294 (20.99)	0.545
Glyburide	747 (3.32)	67 (4.76)	< 0.001	74 (5.25)	69 (4.67)	0.391
Glimepiride	1702 (7.56)	197 (13.95)	< 0.001	160 (11.36)	175 (12.52)	0.251
Chlorpropamide	10 (0.04)	0 (0)	0.3187034	0 (0)	0 (0)	-
Dulaglutide	1015 (4.1)	307 (12.49)	< 0.001	231 (11.12)	208 (10.01)	0.246
Liraglutide	801 (3.56)	144 (10.21)	< 0.001	120 (9)	120 (8.57)	0.622
Lixisenatide	23 (0.1)	6 (0.41)	< 0.001	7 (0.48)	7 (0.48)	1.000
Semaglutide	583 (2.59)	126 (8.91)	< 0.001	105 (7.51)	92 (6.55)	0.225
Pioglitazone	830 (3.69)	104 (7.36)	< 0.001	90 (6.4)	89 (6.31)	0.899
Rosiglitazone	59 (0.26)	6 (0.41)	0.165	7 (0.48)	7 (0.48)	1
Teplizumab	0 (0)	0 (0)	-	0 (0)	0 (0)	-
Tirzepatide	29 (0.13)	6 (0.41)	< 0.01	7 (0.48)	7 (0.48)	1
Tolazamide	0 (0)	0 (0)	-	0 (0)	0 (0)	-
Tolbutamide	9 (0.04)	0 (0)	0.319	0 (0)	0 (0)	-
**Labs**						
Alanine aminotransferase	47.59 ± 76.1	47.7 ± 96.33	0.965	45.25 ± 65.54	47.92 ± 96.65	0.459
Albumin	3.84 ± 0.63	3.96 ± 0.57	< 0.001	3.85 ± 0.62	3.96 ± 0.57	< 0.001
Alkaline phosphatase	105.84 ± 76.39	103.43 ± 74.05	0.315	112.35 ± 84.16	103.62 ± 74.26	0.011
Ammonia	149.42 ± 2061.59	39.59 ± 26.49	0.656	41.16 ± 30.7	39.61 ± 26.69	0.754
Aspartate aminotransferase	46.67 ± 85.09	43.13 ± 62.75	0.182	47.33 ± 106.21	43.27 ± 62.94	0.285
Direct Bilirubin	0.42 ± 1.29	0.35 ± 0.81	0.168	0.33 ± 0.74	0.35 ± 0.81	0.621
Indirect Bilirubin	0.63 ± 0.83	0.58 ± 0.41	0.539	0.51 ± 0.26	0.59 ± 0.41	0.087
Total Bilirubin	0.81 ± 1.34	0.75 ± 0.88	0.157	0.78 ± 1	0.75 ± 0.88	0.533
BMI	33.21 ± 7.48	33.75 ± 7.2	0.057	33.35 ± 7.2	33.74 ± 7.2	0.299
Body weight	208.85 ± 58.84	210.73 ± 59.45	0.333	210.22 ± 57.2	210.77 ± 59.48	0.835
Calcium	9.2 ± 0.77	9.32 ± 0.65	< 0.001	9.23 ± 0.68	9.32 ± 0.65	< 0.01
Creatinine	0.92 ± 2.44	0.95 ± 0.38	0.656	0.94 ± 1.04	0.95 ± 0.38	0.829
Gamma glutamyl transferase	160.8 ± 268.66	138.5 ± 212.96	0.214	171.01 ± 281.18	138.64 ± 214.04	0.170
Estimated Glomerular Filtration Rate	83.56 ± 28.91	78.29 ± 28.39	< 0.001	80.72 ± 28.14	78.45 ± 28.38	0.058
Glucose	151.01 ± 74.91	176.45 ± 83.52	< 0.001	154.79 ± 90.74	176.31 ± 83.35	< 0.001
Hemoglobin A1c	7.2 ± 1.9	8.11 ± 2.03	< 0.001	7.36 ± 1.85	8.11 ± 2.03	< 0.001
INR	1.25 ± 1.54	1.14 ± 0.39	0.035	1.25 ± 1.3	1.14 ± 0.4	0.022
Platelets	208.63 ± 93.44	208.46 ± 90.16	0.952	204.96 ± 91.22	208.42 ± 90.1	0.376
Urea nitrogen	15.34 ± 8.86	17.16 ± 8.83	< 0.001	16.11 ± 8.95	17.1 ± 8.8	0.011
**Liver Disease Severity**						
FIB-4	1.90	1.81	-	2.08	1.81	-
MELD-Na	7.35	6.34	-	7.41	6.32	-

Baseline characteristics for type 2 diabetes (T2DM) patients with cirrhosis. Values are n (%) or Mean ± SD

*BMI, Body Mass Index; ALT, Alanine Aminotransferase; AST, Aspartate Aminotransferase; FIB-4, Fibrosis-4; INR, International Normalized Ratio; Hemoglobin A1c, Hb1Ac; MELD-Na, Model for End-Stage Liver Disease; SGLT2-I, Sodium glucose cotransporter-2 inhibitors*

TriNetX provides real-time live analytics on its platform (TriNetX LLC). The platform analytics balances each patient from the smaller cohorts by choosing matches from the larger cohort through the 1:1 greedy-nearest-neighbor approach using logistic regression from the scikit-learn package in Python version 3.7 with Scipy 1.5.2. This approach used a caliper of 0.1 pooled standard deviations and randomization of the order of records with fixed seeding to increase the reproducibility of matching. The R survival package v3.2-3 was used for Kaplan Meier analysis. Cox proportional hazard models were used to estimate hazard ratios with 95% confidence intervals using the same package. Patients with any prior outcomes of interest before the inception window of receiving mono or dual therapy were excluded from the analysis. Statistical significance was considered with a 2-sided *P* value of 0.05 or less.

## Results

After applying exclusion criteria, we identified patients with type 2 diabetes and cirrhosis, including 22,515 on monotherapy and 1,411 on dual therapy (Fig. 1). Patients on SGLT2-I consisted of 863 patients on empagliflozin, 395 on dapagliflozin, 175 on canagliflozin, and 12 on ertugliflozin. We also identified 3,358 patients with type 2 diabetes and MASH cirrhosis, of whom 2,820 were on monotherapy, and 538 were on dual therapy (Supplementary Fig. 1).

The baseline characteristics of type 2 diabetes patients with MASH cirrhosis before and after PS-matching are detailed in Supplementary Table 6. The PS-matched cohorts were similar overall, though we noted some differences, including higher hemoglobin A1c (7.36% vs. 8.11%, *p* < 0.001) in the dual therapy group that persisted in the MASH groups (A1c 7.61% vs. 8.06%, *p* < 0.001). Supplemental Table 7 outlines baseline cohort diagnoses associated with cirrhosis etiology.

### Primary outcome-mortality

**Fig. 2:**
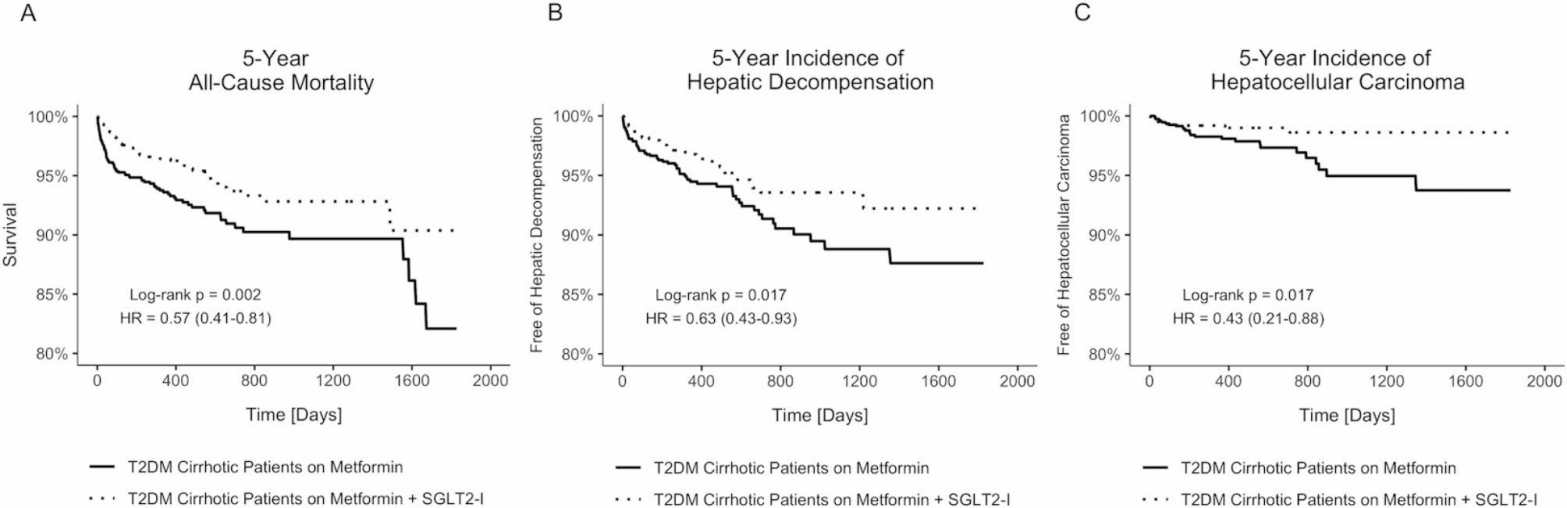
Comparison of 5-Year All-Cause Mortality, Composite Incidence of Hepatic Decompensation, and Hepatocellular Carcinoma (HCC) between Cohorts; Kaplan Meier probabilities values are percent free of death (A), composite hepatic decompensation (B), and hepatocellular carcinoma (C). P values indicate P Log-rank Test. T2DM, Type 2 Diabetes; SGLT2-I, Sodium glucose cotransporter-2 inhibitors.; HR, Hazard Ratio; CI, Confidence Interval

After PS-matching, we found that the dual metformin and SGLT2-I group had decreased risk for mortality (HR 0.57, 95%CI 0.41–0.81) compared to those treated with mono metformin and SGLT2-I therapy (Table 2; Fig. 2A). By sex, men on dual therapy had a decreased risk for mortality (HR 0.62, 95%CI 0.4–0.96). Women on dual treatment had less than half the risk of mortality (HR 0.46, 95%CI 0.26–0.79) compared to women on monotherapy. Likewise, White patients on metformin and SGLT2-I had less than half the mortality risk (HR 0.39, 95%CI 0.26–0.60) relative to White patients on monotherapy. Non-Hispanic patients on dual therapy had more than half the mortality risk (HR 0.40, 95%CI 0.27–0.62) compared to Non-Hispanic patients on monotherapy. Older patients aged 60–80 had decreased mortality risk (HR 0.58, 95%CI 0.38–0.88) when on dual compared to monotherapy. Between mono and dual therapy cohorts, no significant differences in mortality risk and survival rate were observed for Non-Whites, Hispanic patients, the younger 39–59 age group, and type 2 diabetes patients with MASH cirrhosis.

**Table 2:** 5-Year Risk of Mortality in T2DM Patients with Cirrhosis

		Kaplan Meier Estimates			
**5-Year Survival Rate (%)**		After Propensity Score Matching			
		Metformin	Metformin + SGLT2 I	P	HR (95%CI)
All		82.09	90.38	0.002	0.57 (0.41–0.81)
Subgroup ^a^					
Men		77.76	85.09	0.031	0.62 (0.4–0.96)
Women		84.52	94.8	< 0.01	0.46 (0.26–0.79)
White		80.08	93.96	< 0.001	0.39 (0.26–0.6)
Non-White		83.22	82.58	0.468	0.7 (0.27–1.85)
Hispanic		96.4	98.18	0.464	0.53 (0.1–2.94)
Non-Hispanic		75.58	93.83	< 0.001	0.4 (0.27–0.62)
Age 39–59		92.73	94.26	0.305	0.68 (0.32–1.43)
Age 60–80		82.66	88.62	0.011	0.58 (0.38–0.88)
MASH ^b^		93.82	89.89	0.746	0.82 (0.25–2.7)

^a^ Type 2 diabetes mellitus (T2DM) patients with cirrhosis treated with metformin and sodium glucose cotransporter-2 inhibitors (SGLT2-I) were further divided into demographic subgroups and propensity score matched to the Metformin group by sex, race, ethnicity, and age groups

^b^ T2DM patients with non-alcoholic steatohepatitis (MASH) Cirrhosis treated with metformin and SGLT2-I were propensity score matched to T2DM patients with MASH cirrhosis on metformin (n = 2,820)

K-M probabilities values are percent free of death. P values indicate P Log-rank Test

*CI, Confidence Interval; HR, Hazard Ratio; K-M, Kaplan Meier*

### Secondary outcome-composite hepatic decompensation

Upon PS-matching, we found that the composite risk of becoming decompensated was decreased in type 2 diabetes patients with cirrhosis on dual therapy (HR 0.63, 95%CI 0.43–0.93) compared to monotherapy patients (Table 3; Fig. 2B). White patients on dual treatment had half the risk of decompensation (HR 0.49, 95%CI 0.31–0.76) compared to White patients on monotherapy. Conversely, there were no observed differences between mono and dual therapy for Non-White patients. Reduced hepatic decompensation occurrence was seen in patients aged 60–80 on metformin and SGLT2-I therapy (HR 0.53, 95%CI 0.33–0.85). This difference was not seen between mono and dual therapy treated younger patients aged 39–59. We did not find a statistically significant difference in decompensation events between treatment cohorts by sex, ethnicity, or in the subset of patients with cirrhosis due to MASH.

**Table 3:** 5-Year Risk of Hepatic Decompensation in T2DM Patients with Cirrhosis

		Kaplan Meier Estimates			
**5-Year Absence of Hepatic Decompensation (%)**		After Propensity Score Matching			
		Metformin	Metformin + SGLT2-I	P	HR (95%CI)
All		87.62	92.22	0.017	0.63 (0.43–0.93)
Subgroup ^a^					
Men		87.42	87.78	0.477	0.83 (0.51–1.37)
Women		88.72	96.02	0.086	0.57 (0.29–1.09)
White		87.43	92.51	< 0.01	0.49 (0.31–0.76)
Non-White		93.05	94.31	0.105	0.43 (0.15–1.23)
Hispanic		91.82	98.4	0.230	0.39 (0.08–1.92)
Non-Hispanic		87.84	91.53	0.185	0.73 (0.46–1.16)
Age 39–59		94.86	95.08	0.708	1.18 (0.51–2.7)
Age 60–80		85.84	90.98	< 0.01	0.53 (0.33–0.85)
MASH ^b^		95.39	97.43	0.124	0.52 (0.22–1.22)

^a^ Type 2 diabetes mellitus (T2DM) patients with cirrhosis treated with metformin and sodium glucose cotransporter-2 inhibitors (SGLT2-I) were further divided into demographic subgroups and propensity score matched to the Metformin group by sex, race, ethnicity, and age groups

^b^ T2DM patients with non-alcoholic steatohepatitis (MASH) Cirrhosis treated with metformin and SGLT2-I were propensity score matched to T2DM patients with MASH cirrhosis on metformin (n = 2,820)

Composite Hepatic Decompensation is defined as having any EMR diagnosis codes for following: ascites, hepatic encephalopathy, and gastric or esophageal variceal bleeding. K-M probabilities values are percent free of death. P values indicate P Log-rank Test

*CI, Confidence Interval; HR, Hazard Ratio; K-M, Kaplan Meier*

### Tertiary outcome-hepatocellular carcinoma

In our matched set of type 2 diabetes patients with cirrhosis, those on dual metformin and SGLT2-I had less than half the risk for developing HCC (HR 0.43, 95%CI 0.21–0.88) compared to those on mono metformin therapy (Table 4; Fig. 2C). White patients on dual therapy had a reduced risk of HCC occurrence (HR 0.44, 95%CI 0.21–0.93) compared to White patients on monotherapy. No difference was observed between treatment groups for Non-White patients. Non-Hispanic patients on dual metformin and SGLT2-I therapy had a decreased risk of developing HCC (0.46, 95%CI 0.22–0.97). Conversely, there was no difference in HCC risk and occurrence between Hispanic patients on mono and dual therapy. Further, we did not find significant differences in the occurrence of HCC between mono and dual therapy cohorts by sex and age groups or in patients with MASH cirrhosis.

**Table 4:** 5-Year Risk of Hepatocellular Carcinoma in T2DM Patients with Cirrhosis

		Kaplan Meier Estimates			
**5-Year Absence of Hepatocellular Carcinoma (%)**		After Propensity Score Matching			
		Metformin	Metformin + SGLT2-i	P	HR (95%CI)
All		93.75	98.61	0.017	0.43 (0.21–0.88)
Subgroup ^a^					-
Men		86.91	99.64	0.051	0.24 (0.05–1.14)
Women		85.02	97.72	0.106	0.52 (0.23–1.16)
White		92.71	98.18	0.029	0.44 (0.21–0.93)
Non-White		96.87	99.49	0.100	0.2 (0.02–1.69)
Hispanic		95.54	99.17	0.385	0.38 (0.04–3.7)
Non-Hispanic		93.32	98.18	0.037	0.46 (0.22–0.97)
Age 39–59		95.21	99.44	0.134	0.31 (0.06–1.56)
Age 60–80		97.18	98.64	0.051	0.4 (0.16–1.03)
MASH ^b^		93.61	98.72	0.120	0.08 (0.01–0.62)

^a^ Type 2 diabetes mellitus (T2DM) patients with cirrhosis treated with metformin and sodium glucose cotransporter-2 inhibitors (SGLT2-I) were further divided into demographic subgroups and propensity score matched to the Metformin group by sex, race, ethnicity, and age groups

^b^ T2DM patients with non-alcoholic steatohepatitis (MASH) Cirrhosis treated with metformin and SGLT2-I were propensity score matched to T2DM patients with MASH cirrhosis on metformin (n = 2,820)

K-M probabilities values are percent free of death. P values indicate P Log-rank Test

*CI, Confidence Interval; HR, Hazard Ratio; K-M, Kaplan Meier*

## Discussion

In our multicenter, retrospective PS-matched study of type 2 diabetes patients with cirrhosis, we found a possible associated benefit in reducing both mortality and complications of cirrhosis, such as hepatic decompensation and HCC, when patients were on dual therapy with both metformin and an SGLT2-I agent compared to those on metformin monotherapy. Dual treatment did not reduce mortality, decompensated cirrhosis, or HCC incidence in the MASH cirrhosis subset.

While SGLT2-I has been known for its direct glucose-lowering effects in diabetic patients [1], growing evidence supports its use in patients with chronic liver diseases. In pre-clinical models of MASH and MASLD, SGLT2-I treatment reduced HbA1c, steatosis, liver triglycerides, liver apoptosis, and inflammatory molecules [16, 17]. In clinical studies, patients with non-cirrhotic, but biopsy-confirmed MASH treated with an SGLT2-I had histological improvement, decreased lobular inflammation, fibrosis, and reduced serum AST and ALT [18, 19]. The evidence supports the hypothesis that novel antihyperglycemics, such as SGLT2-I, may also benefit type 2 diabetes patients with cirrhosis. Still, recent findings have been limited to pre-clinical, observational, and small retrospective studies. However, our investigation drew upon multicenter data to directly compare monotherapy and dual therapy groups and provides compelling evidence for the use of SGLT2-I in patients with type 2 diabetes and cirrhosis.

Our study hypothesizes that the mortality benefit of patients on dual metformin and SGLT2-I therapy can be attributed to SGLT2-I glucose-lowering actions and their pleiotropic effects. Inhibiting SGLT2 decreases glucose reabsorption in proximal renal tubules and control of glycemic indices, which is reflected in decreased Hb1Ac and increased insulin sensitivity. Studies have demonstrated that long-standing hyperglycemia promotes liver damage and could potentially increase complications such as hepatic encephalopathy in patients with cirrhosis [8, 20]. The use of SGLT2-I has been established in type 2 diabetes comorbidities. Cardiovascular outcomes trials (CVOTs) [21, 22] have shown that SGLT2-I reduces cardiovascular-related (CV) deaths, potentially through mechanisms such as diuresis, reduced preload and afterload, and improved blood pressure. Renal injury complications necessitating dialysis are found in type 2 diabetes and cirrhosis patients [23]. Further, end-stage renal disease (ESRD) has been shown to increase 3-year mortality in cirrhosis patients by 2-fold [24]. SGLT2-I can mitigate multiple risk factors for renal impairment and is safely used in those with chronic kidney disease stage 3 and greater [25, 26], but because metformin was a prerequisite for cohort construction, we excluded patients with ESRD, and therefore, our results do not extend to this population.

Our study demonstrates that using metformin and SGLT2-I is associated with reduced decompensated cirrhosis. The SGLT2-I may target neurohumoral pathways and alleviate the renin-angiotensin-aldosterone system (RAAS), an essential system in the pathogenesis of portal hypertension [12]. Clinically significant portal hypertension marks the formation of portal-systemic shunts that are associated with developing variceal bleeding, hepatic encephalopathy, and ascites [27]. Therefore, portal hypertension accelerates the progression from compensated to decompensated cirrhosis, a state associated with a worse prognosis [28]. Additional studies are required to elucidate whether SGLT2-Is can reduce portal hypertension [29, 30] and its complications, such as ascites.

A previous study compared SGLT2-I to DPP-4 inhibitor treatment in patients with cirrhosis and type 2 diabetes who were on metformin; this study found a decreased risk of mortality in those with SGLT2-I use but no difference in the risk of developing ascites compared to DPP-4 inhibitor users [30]. In contrast, smaller studies show that the use of SGLT2-I improves ascites and peripheral edema outcomes in type 2 diabetes patients with cirrhosis [31]. In a separate sub-analysis, our study investigated whether those on dual metformin and SGLT2-I therapy had decreased risk for ascites; however, given small sample sizes, SGLT2-I utility in ascites reduction was indeterminant. Nevertheless, data exists to support the use of these therapeutics. The antagonism of sodium-glucose cotransporter 2 in the proximal tubule in the nephron leads to natriuresis and decreased activation of RAAS, thus reducing salt and water retention. However, further investigation is required to ascertain the role of SGLT2-I in treating cirrhosis complications such as ascites.

HCC severely worsens a patient’s prognosis and is an independent risk factor for mortality in decompensated cirrhosis patients [32]. While current retrospective studies show that SGLT2-I improves survival in patients with type 2 diabetes and HCC [34], few have examined whether SGLT2-I can reduce incidences of HCC in patients with underlying cirrhosis. In vitro studies demonstrate that canagliflozin induces apoptosis, reduces metabolic reprogramming to glycolysis, and inhibits glucose uptake, which prevents cellular proliferation in hepatocellular carcinoma models [33, 34]. In conjunction with these studies, we show a potential benefit in using SGLT2-I to reduce the occurrence of HCC and to improve mortality and morbidity in type 2 diabetes patients with cirrhosis.

In our subset of type 2 diabetes patients with MASH cirrhosis, our study did not find differences in outcomes between mono- and dual-therapy-treated cohorts. Despite not corroborating current SGLT2-I studies in MASH patients without cirrhosis, there are still implications for the utility of SGLT2-I in chronic liver diseases. Empagliflozin has been shown to reduce liver fat, fibrosis, and BMI among type 2 diabetes patients with MASH [35]. To our knowledge, few clinical trials are investigating the use of SGLT2-I in cirrhosis patients. Current clinical trials are evaluating the efficacy and safety of dapagliflozin and empagliflozin in patients with cirrhosis with recurrent ascites (NCT05014594 and NCT05013502). Overall, there is a need for further prospective studies as SGLT2-I may benefit type 2 diabetes patients with cirrhosis.

### Limitations

Our study has certain limitations. Because TriNetX does not provide imaging or biopsy data, we relied on ICD-10 code diagnoses for cohort construction. The cirrhosis diagnosis standard requires a liver biopsy or radiological testing through magnetic resonance imaging (MRI) or computed tomography (CT). Therefore, the counts for patients with type 2 diabetes and cirrhosis may be low. Undercounting may also affect our MASH cirrhosis subset since there was no official ICD-10 code for MASH cirrhosis before 2021, and current literature [36] cites ICD-10 codes K76.0 and K75.8 for patients who have been diagnosed with MASH. Due to the small population size of our MASH cirrhosis patients, our study outcomes are indeterminant.

A second limitation of our study is the small size of our Non-White and Hispanic patient cohorts. While we did not observe mortality benefit, decreased hepatic decompensation risks, or reduced HCC occurrence associated with metformin and SGLT2-I dual therapy use in these demographic sub-groups, our outcomes are likewise indeterminant for these groups due to the small population size.

Since online platform analytics on TriNetX utilizes aggregate patient data, we could not calculate FIB-4 or MELD-Na scores to predict mortality or determine fibrosis severity at the single patient level. These scores were thus calculated in aggregate across our cohorts of interest. Based on the same limitation, we could not look at specific causes of mortality given the privacy regulations of TriNetX, and therefore, all-cause mortality was used as our primary outcome. Over 75% of mono and dual therapy cohort patients have an ICD-10 diagnosis for hypertensive and metabolic diseases, suggesting its role in patient mortality.

Our study defined composite hepatic decompensation as having any diagnoses for ascites, esophageal or gastric variceal bleeding, and hepatic encephalopathy. Because other studies may have chosen to include different cirrhotic complications, such as spontaneous bacterial peritonitis (SBP) and hepatorenal syndrome, in their study definitions, more confirmatory studies are needed. Though we did not conduct sensitivity analyses, previous studies demonstrate ICD-10 codes as having high positive predictive values for patients with cirrhosis and its related complications [37, 38]. Studies validating the use of ICD-10 codes to identify patients with cirrhosis within the TriNetX network have yet to be published.

## Conclusion

In conclusion, our study suggests a role for SGLT2-I in the setting of diabetes mellitus and cirrhosis. We demonstrated an associated mortality benefit and a possible benefit in reducing complications of cirrhosis in type 2 diabetes patients with cirrhosis on dual metformin and SGLT2-I compared to metformin monotherapy. We look forward to prospective studies that can clarify the role of SGLT2-I in this population.

### Electronic supplementary material

Below is the link to the electronic supplementary material.

Supplementary Material 1

## Data Availability

The data that supports this study are available on TriNetX. Given the restrictions and licensing involved with the current study, the data is not publicly available. However, data are available upon reasonable request to the corresponding author and permission from the TriNetX server.

## Abbreviations

ALT: Alanine transaminase
AST: Aspartate transaminase
CI: Confidence interval
CPT: Current procedural terminology
CV: Cardiovascular related
DPP-4: Dipeptidyl Peptidase-4
EMR: Electronic medical record
ESRD: End-Stage Renal Disease
HF: Heart Failure
HCC: Hepatocellular Carcinoma
HE: Hepatic Encephalopathy
ICD-10: International classification of diseases 10
LOINC: logistical observation identifiers names and codes
MASLD: Metabolic dysfunction-associated steatotic liver disease
MASH: Metabolic dysfunction – associated steatohepatitis
PS: Propensity score
RAAS: Renin-Angiotensin Aldosterone System
SD: Standard deviation
SGLT2-I: Sodium glucose cotransporter-2 inhibitors
TIPS: Transvenous intrahepatic portosystemic shunt
T2DM: Type 2 diabetes mellitus
