# Supplementary material for: Reduced mortality and morbidity associated with metformin and SGLT2 inhibitor therapy in patients with type 2 diabetes mellitus and cirrhosis
Source: BMC Gastroenterol. 2023 Dec 19;23:450. doi: 10.1186/s12876-023-03085-8 (PMC10731715; doi:10.1186/s12876-023-03085-8)
Supplement: Supplementary file 1 — Supplementary Material 1 [file 12876_2023_3085_MOESM1_ESM.docx]

**Supplementary Material**

**Supplemental Table 1.** Patient Cohort Count

| **Patient Count** | Before Propensity Score Matching | |  | After Propensity Score Matching | |
| --- | --- | --- | --- | --- | --- |
|  | Metformin | Metformin + SGLT2-I |  | Metformin | Metformin + SGLT2-I |
| All | 22515 | 1411 |  | 1403 | 1403 |
| Demographic Subgroup |  |  |  |  |  |
| Men | 10368 | 688 |  | 678 | 678 |
| Women | 11645 | 707 |  | 695 | 695 |
| White | 15267 | 985 |  | 979 | 979 |
| Non-White | 4121 | 242 |  | 223 | 223 |
| Hispanic | 2570 | 176 |  | 157 | 157 |
| Non-Hispanic | 14816 | 980 |  | 972 | 972 |
| Age 39-59 | 6180 | 458 |  | 445 | 445 |
| Age 60-80 | 12835 | 841 |  | 837 | 837 |
| NASH | 2820 | 538 |  | 535 | 535 |

Patient cohort count was reported before and after propensity score matching.

**Supplemental Figure 1.** Cohort Construction of T2DM Patients with NASH Cirrhosis

**
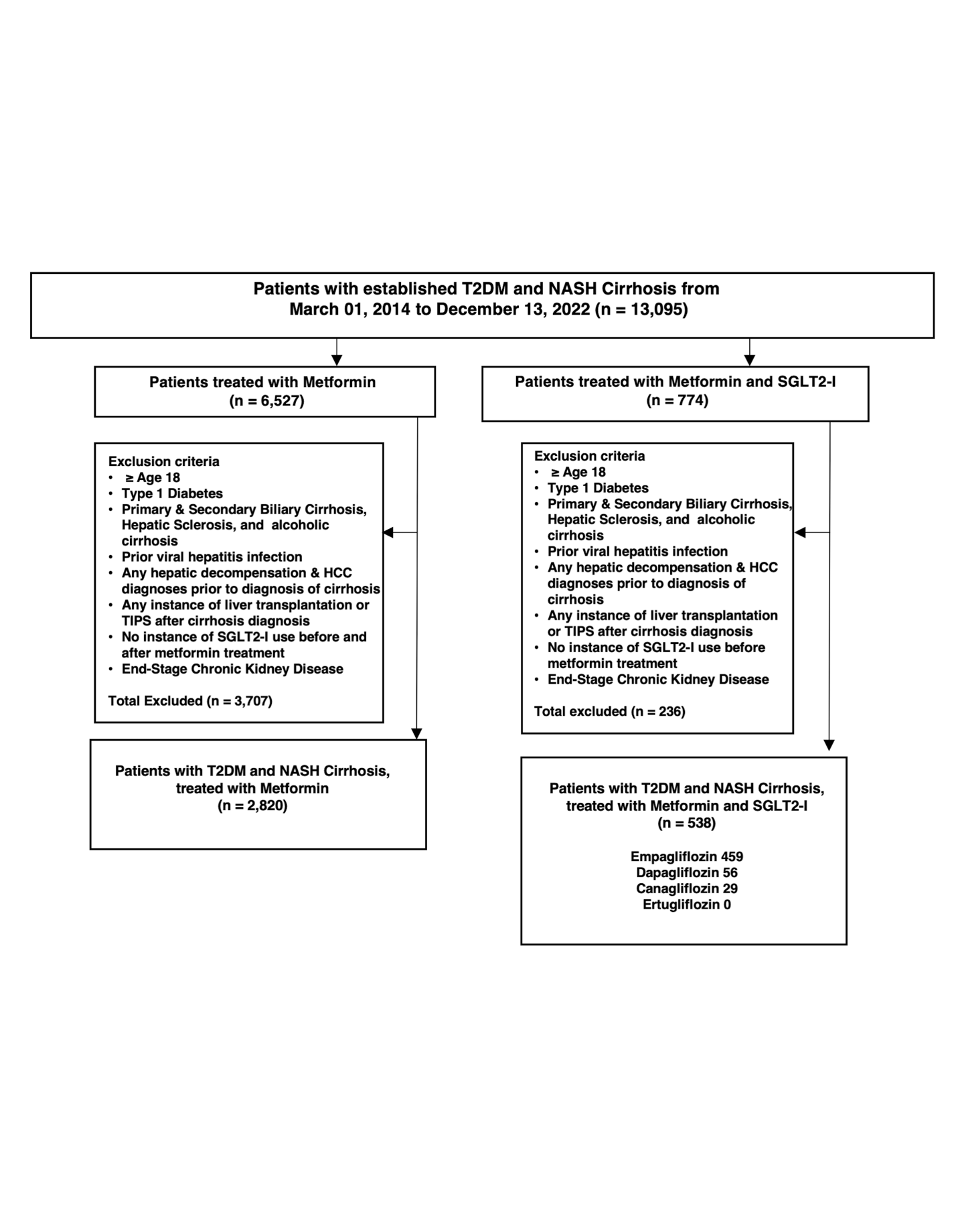
**

Cohort Construction. Inclusion and exclusion criteria for patient cohort selection were based on ICD-10 coding, Current Procedural Terminology, and RxNorm terminology.

**Supplemental Table 2.** Cohort Baseline FIB-4 Scores

|  | Before PS-Matching Characteristics | |  | After PS-Matching Characteristics | |
| --- | --- | --- | --- | --- | --- |
|  | Metformin | Metformin + SGLT2-I |  | Metformin | Metformin + SGLT2-I |
|  | FIB-4 | FIB-4 |  | FIB-4 | FIB-4 |
| All | 1.90 | 1.81 |  | 2.08 | 1.81 |
| Subgroup^†^ |  |  |  |  |  |
| Men | 2.31 | 1.93 |  | 2.10 | 1.96 |
| Women | 1.60 | 1.74 |  | 1.76 | 1.75 |
| White | 1.93 | 1.90 |  | 2.09 | 1.90 |
| Non-White | 1.82 | 1.58 |  | 1.83 | 1.58 |
| Hispanic | 1.70 | 1.89 |  | 1.72 | 1.95 |
| Non-Hispanic | 1.87 | 1.85 |  | 2.07 | 1.86 |
| Age 39-59 | 1.47 | 1.43 |  | 1.56 | 1.43 |
| Age 60-80 | 2.33 | 2.10 |  | 2.20 | 2.10 |
| NASH^‡^ | 1.76 | 1.63 |  | 1.71 | 1.64 |

FIB-4 scores were calculated for each cohort based on average lab values reported by TriNetX.

**Supplemental Table 3.** Cohort Baseline MELD-Na Scores

|  | Before PS-Matching Characteristics | |  | After PS-Matching Characteristics | |
| --- | --- | --- | --- | --- | --- |
|  | Metformin | Metformin + SGLT2-I |  | Metformin | Metformin + SGLT2-I |
|  | MELD-Na | MELD-Na |  | MELD-Na | MELD-Na |
| All | 7.35 | 6.34 |  | 7.41 | 6.32 |
| Subgroup^†^ |  |  |  |  |  |
| Men | 9.18 | 8.44 |  | 8.56 | 8.42 |
| Women | 5.58 | 4.07 |  | 5.93 | 4.11 |
| White | 7.38 | 6.19 |  | 9.01 | 6.17 |
| Non-White | 7.36 | 6.44 |  | 6.43 | 6.21 |
| Hispanic | 7.05 | 5.19 |  | 6.65 | 5.21 |
| Non-Hispanic | 7.44 | 6.35 |  | 8.61 | 6.35 |
| Age 39-59 | 6.20 | 5.28 |  | 8.74 | 4.78 |
| Age 60-80 | 7.94 | 6.95 |  | 7.95 | 6.94 |
| NASH^‡^ | 5.52 | 4.24 |  | 6.32 | 4.21 |

MELD-Na scores were calculated for each cohort based on average lab values reported by TriNetX.

**Supplemental Table 4.** Study Definitions

| **Diagnosis** | **ICD-10 Code For Inclusion** |
| --- | --- |
| Type 2 Diabetes | E11 |
| Cirrhosis | K70.3, K74, K74.6, K74.2 |
| Non-alcoholic steatohepatitis NASH | K76.0, K75.8 |
| Ascites | R18, R18.8 |
| Varices | I85.0, I85.1, I86.4, I85.11 |
| Hepatic Encephalopathy | G93.40, G93.41, G93.49, K72.91 |
| **Diagnosis** | **ICD-10 Code For Exclusion** |
| Type 1 Diabetes | E10 |
| Primary and Secondary Biliary Cirrhosis and Hepatic Sclerosis | K74.3, K74.1, K74.4 |
| Viral Hepatitis | B17.1, B18.2, B19.2, B16.0, B17.0, B18.0, B19.1, B17.9, B18.1, B19.9 |
| End Stage Chronic Kidney Disease (Stage 4 & 5) | N18.4, N18.5 |
| Hepatocellular Carcinoma | C22.0, C22.8 |
| **Procedures** | **Current Procedural Terminology** |
| transvenous intrahepatic portosystemic shunt (TIPS) | 06180ZY, 37183 |
| Liver Transplantation | 47135 |
| **Medications** | **RxNorm** |
| Metformin | 6809 |
| SGLT2-I | 1992672 ertugliflozin, 1373458 canagliflozin, 1488564 dapagliflozin, 1545653 empagliflozin |
| **Demographics** |  |
| Non-White | Non-White is defined as Black or African American, Asian, Native Hawaiian or Pacific Islander, Alaskan Native or American Indian. |
| Age | Defined as Age of Patients at the index of receiving mono or dual treatment |

Inclusion and exclusion criteria for patient cohort selection was based on ICD-10 coding, Current Procedural Terminology, and RxNorm terminology. For details review Materials and Methods.

**Supplementary Table 5.** Full, Before & After Propensity Score Matched Baseline Characteristics for T2DM patients with Cirrhosis

| T2DM Cirrhosis | | | | | | | | |
| --- | --- | --- | --- | --- | --- | --- | --- | --- |
|  |  | Before Propensity Score Matching | | |  | After Propensity Score Matching | | |
| Characteristic ID | Characteristic Name | Metformin | Metformin + SGLT2-I | P |  | Metformin | Metformin + SGLT2-I | P |
| AI | Age at Index | 58.56 ± 14.41 | 60.35 ± 11.67 | < 0.001 |  | 60.55 ± 12.88 | 60.34 ± 11.66 | 0.642 |
| Age | Current Age | 62.04 ± 14.37 | 62.15 ± 11.59 | 0.785 |  | 62.39 ± 12.82 | 62.14 ± 11.58 | 0.584 |
| M | Male | 10368 (46.05) | 688 (48.76) | 0.048 |  | 670 (47.75) | 684 (48.75) | 0.597 |
| F | Female | 11645 (51.72) | 707 (50.11) | 0.239 |  | 711 (50.68) | 703 (50.11) | 0.763 |
| UN | Unknown Gender | 502 (2.23) | 16 (1.13) | < 0.01 |  | 22 (1.57) | 16 (1.14) | 0.327 |
| 2106-3 | White | 15267 (67.81) | 985 (69.81) | 0.118 |  | 1025 (73.06) | 980 (69.85) | 0.060 |
| 1002-5 | American Indian or Alaska Native | 214 (0.95) | 10 (0.71) | 0.360 |  | 10 (0.71) | 10 (0.71) | 1.000 |
| 2028-9 | Asian | 599 (2.66) | 36 (2.55) | 0.805 |  | 26 (1.85) | 36 (2.57) | 0.199 |
| 2054-5 | Black or African American | 3278 (14.56) | 195 (13.82) | 0.444 |  | 177 (12.62) | 194 (13.83) | 0.343 |
| 2076-8 | Native Hawaiian or Other Pacific Islander | 30 (0.13) | 10 (0.71) | < 0.001 |  | 10 (0.71) | 10 (0.71) | 1.000 |
| 2131-1 | Unknown Race | 3127 (13.89) | 184 (13.04) | 0.371 |  | 166 (11.83) | 183 (13.04) | 0.331 |
| 2135-2 | Hispanic or Latino | 2570 (11.42) | 176 (12.47) | 0.226 |  | 170 (12.12) | 174 (12.4) | 0.818 |
| 2186-5 | Not Hispanic or Latino | 14816 (65.81) | 980 (69.45) | < 0.01 |  | 992 (70.71) | 974 (69.42) | 0.458 |
| UN | Unknown Ethnicity | 5129 (22.78) | 255 (18.07) | < 0.001 |  | 241 (17.18) | 255 (18.18) | 0.488 |
| N17-N19 | Acute kidney failure and chronic kidney disease | 3813 (16.93) | 437 (30.97) | < 0.001 |  | 427 (30.44) | 431 (30.72) | 0.870 |
| F50-F59 | Behavioral syndromes associated with physiological disturbances and physical factors | 1194 (5.3) | 111 (7.87) | < 0.001 |  | 101 (7.2) | 111 (7.91) | 0.475 |
| K00-K95 | Diseases of the digestive system | 16811 (74.67) | 1271 (90.08) | < 0.001 |  | 1247 (88.88) | 1263 (90.02) | 0.325 |
| E00-E89 | Endocrine, nutritional and metabolic diseases | 19630 (87.19) | 1334 (94.54) | < 0.001 |  | 1306 (93.09) | 1326 (94.51) | 0.117 |
| I50 | Heart failure | 2743 (12.18) | 323 (22.89) | < 0.001 |  | 316 (22.52) | 316 (22.52) | 1.000 |
| I10-I16 | Hypertensive diseases | 15006 (66.65) | 1134 (80.37) | < 0.001 |  | 1086 (77.41) | 1127 (80.33) | 0.058 |
| I20-I25 | Ischemic heart diseases | 4757 (21.13) | 482 (34.16) | < 0.001 |  | 477 (34) | 475 (33.86) | 0.936 |
| F10-F19 | Mental and behavioral disorders due to psychoactive substance use | 6860 (30.47) | 472 (33.45) | 0.018 |  | 446 (31.79) | 469 (33.43) | 0.354 |
| E70-E88 | Metabolic disorders | 14180 (62.98) | 1137 (80.58) | < 0.001 |  | 1131 (80.61) | 1129 (80.47) | 0.924 |
| E13 | Other specified diabetes mellitus | 948 (4.21) | 84 (5.95) | < 0.01 |  | 81 (5.77) | 83 (5.92) | 0.872 |
| E65-E68 | Overweight, obesity and other hyperalimentation | 9225 (40.97) | 782 (55.42) | < 0.001 |  | 781 (55.67) | 776 (55.31) | 0.849 |
| K76.0 | Fatty (change of) liver, not elsewhere classified | 6004 (28.22) | 614 (43.57) | < 0.001 |  | 566 (40.06) | 575 (40.69) | 0.730 |
| K75.8 | Other specified inflammatory liver diseases | 2251 (10.03) | 321 (22.8) | < 0.001 |  | 306 (21.66) | 274 (19.39) | 0.136 |
| K70.3 | Alcoholic cirrhosis of liver | 1148 (5.1) | 106 (7.54) | < 0.001 |  | 99 (7.08) | 96 (6.86) | 0.824 |
| B18.2 | Chronic viral hepatitis C | 3005 (13.35) | 196 (13.88) | 0.533 |  | 179 (12.67) | 188 (13.3) | 0.615 |
| B18.1 | Chronic viral hepatitis B without delta-agent | 23 (0.11) | 0 (0) | 0.179 |  | 0 (0) | 0 (0) | NA |
| B18.0 | Chronic viral hepatitis B with delta-agent | 495 (2.22) | 36 (2.15) | 0.854 |  | 28 (1.98) | 30 (2.12) | 0.791 |
| K74.6 | Other and unspecified cirrhosis of liver | 5617 (24.95) | 790 (56.31) | < 0.001 |  | 689 (48.76) | 686 (48.55) | 0.910 |
| K75.4 | Autoimmune hepatitis | 225 (1) | 22 (1.32) | 0.218 |  | 24 (1.7) | 20 (1.42) | 0.543 |
| E83.11 | Hemochromatosis | 209 (0.93) | 19 (1.14) | 0.401 |  | 15 (1.06) | 14 (0.99) | 0.852 |
| E88.01 | Alpha-1-antitrypsin deficiency | 39 (0.18) | 10 (0.6) | < 0.001 |  | 10 (0.71) | 10 (0.71) | 1 |
| 1003143 | Surgery | 15259 (67.77) | 1122 (79.52) | < 0.001 |  | 1069 (76.19) | 1114 (79.4) | 0.041 |
| 1006964 | Surgical Procedures on the Digestive System | 5828 (25.89) | 559 (39.62) | < 0.001 |  | 552 (39.34) | 554 (39.49) | 0.938 |
| CV800 | Ace inhibitors | 8683 (38.56) | 706 (50.03) | < 0.001 |  | 688 (49.04) | 701 (49.96) | 0.624 |
| CV805 | Angiotensin ii inhibitor | 4333 (19.25) | 490 (34.73) | < 0.001 |  | 495 (35.28) | 483 (34.43) | 0.634 |
| CV300 | Antiarrhythmics | 9014 (40.04) | 780 (55.28) | < 0.001 |  | 766 (54.6) | 772 (55.02) | 0.820 |
| BL110 | Anticoagulants | 7875 (34.98) | 669 (47.41) | < 0.001 |  | 637 (45.4) | 663 (47.26) | 0.325 |
| CV490 | Antihypertensives,other | 4042 (17.95) | 360 (25.51) | < 0.001 |  | 354 (25.23) | 356 (25.37) | 0.931 |
| HS503 | Antihypoglycemics | 6241 (27.72) | 582 (41.25) | < 0.001 |  | 580 (41.34) | 576 (41.06) | 0.878 |
| CV350 | Antilipemic agents | 10930 (48.55) | 1007 (71.37) | < 0.001 |  | 996 (70.99) | 1000 (71.28) | 0.868 |
| CV100 | Beta blockers/related | 9079 (40.32) | 778 (55.14) | < 0.001 |  | 756 (53.89) | 772 (55.02) | 0.544 |
| CV200 | Calcium channel blockers | 6017 (26.72) | 508 (36) | < 0.001 |  | 486 (34.64) | 504 (35.92) | 0.477 |
| CV700 | Diuretics | 9774 (43.41) | 799 (56.63) | < 0.001 |  | 784 (55.88) | 793 (56.52) | 0.732 |
| HS509 | Hypoglycemic agents,other | 1722 (7.65) | 381 (27) | < 0.001 |  | 397 (28.3) | 373 (26.59) | 0.310 |
| HS501 | Insulin | 9395 (41.73) | 864 (61.23) | < 0.001 |  | 847 (60.37) | 857 (61.08) | 0.699 |
| HS502 | Oral hypoglycemic agents,oral | 15862 (70.45) | 1249 (88.52) | < 0.001 |  | 1226 (87.38) | 1241 (88.45) | 0.385 |
| BL117 | Platelet aggregation inhibitors | 8412 (37.36) | 767 (54.36) | < 0.001 |  | 757 (53.96) | 760 (54.17) | 0.910 |
| 9044 | Alanine aminotransferase [Enzymatic activity/volume] in Serum, Plasma or Blood | 47.59 ± 76.1 | 47.7 ± 96.33 | 0.965 |  | 45.25 ± 65.54 | 47.92 ± 96.65 | 0.459 |
| 9045 | Albumin [Mass/volume] in Serum, Plasma or Blood | 3.84 ± 0.63 | 3.96 ± 0.57 | < 0.001 |  | 3.85 ± 0.62 | 3.96 ± 0.57 | < 0.001 |
| 9046 | Alkaline phosphatase [Enzymatic activity/volume] in Serum, Plasma or Blood | 105.84 ± 76.39 | 103.43 ± 74.05 | 0.315 |  | 112.35 ± 84.16 | 103.62 ± 74.26 | 0.011 |
| 16362-6 | Ammonia [Moles/volume] in Plasma | 149.42 ± 2061.59 | 39.59 ± 26.49 | 0.656 |  | 41.16 ± 30.7 | 39.61 ± 26.69 | 0.754 |
| 9047 | Aspartate aminotransferase [Enzymatic activity/volume] in Serum or Plasma | 46.67 ± 85.09 | 43.13 ± 62.75 | 0.182 |  | 47.33 ± 106.21 | 43.27 ± 62.94 | 0.285 |
| 9048 | Bilirubin.direct [Mass/volume] in Serum or Plasma | 0.42 ± 1.29 | 0.35 ± 0.81 | 0.168 |  | 0.33 ± 0.74 | 0.35 ± 0.81 | 0.621 |
| 9049 | Bilirubin.indirect [Mass/volume] in Serum or Plasma | 0.63 ± 0.83 | 0.58 ± 0.41 | 0.539 |  | 0.51 ± 0.26 | 0.59 ± 0.41 | 0.087 |
| 9050 | Bilirubin.total [Mass/volume] in Serum, Plasma or Blood | 0.81 ± 1.34 | 0.75 ± 0.88 | 0.157 |  | 0.78 ± 1 | 0.75 ± 0.88 | 0.533 |
| 9083 | BMI | 33.21 ± 7.48 | 33.75 ± 7.2 | 0.057 |  | 33.35 ± 7.2 | 33.74 ± 7.2 | 0.299 |
| 9081 | Body weight | 208.85 ± 58.84 | 210.73 ± 59.45 | 0.333 |  | 210.22 ± 57.2 | 210.77 ± 59.48 | 0.835 |
| 9022 | Calcium [Mass/volume] in Serum, Plasma or Blood | 9.2 ± 0.77 | 9.32 ± 0.65 | < 0.001 |  | 9.23 ± 0.68 | 9.32 ± 0.65 | < 0.01 |
| 9024 | Creatinine [Mass/volume] in Serum, Plasma or Blood | 0.92 ± 2.44 | 0.95 ± 0.38 | 0.656 |  | 0.94 ± 1.04 | 0.95 ± 0.38 | 0.829 |
| 9051 | Gamma glutamyl transferase [Enzymatic activity/volume] in Serum or Plasma | 160.8 ± 268.66 | 138.5 ± 212.96 | 0.214 |  | 171.01 ± 281.18 | 138.64 ± 214.04 | 0.170 |
| 8001 | Glomerular filtration rate/1.73 sq M.predicted [Volume Rate/Area] in Serum, Plasma or Blood by Creatinine-based formula (MDRD) | 83.56 ± 28.91 | 78.29 ± 28.39 | < 0.001 |  | 80.72 ± 28.14 | 78.45 ± 28.38 | 0.058 |
| 9025 | Glucose [Mass/volume] in Serum, Plasma or Blood | 151.01 ± 74.91 | 176.45 ± 83.52 | < 0.001 |  | 154.79 ± 90.74 | 176.31 ± 83.35 | < 0.001 |
| 9037 | Hemoglobin A1c/Hemoglobin.total in Blood | 7.2 ± 1.9 | 8.11 ± 2.03 | < 0.001 |  | 7.36 ± 1.85 | 8.11 ± 2.03 | < 0.001 |
| 9032 | INR in Plasma or Blood | 1.25 ± 1.54 | 1.14 ± 0.39 | 0.035 |  | 1.25 ± 1.3 | 1.14 ± 0.4 | 0.022 |
| 9020 | Platelets [#/volume] in Blood | 208.63 ± 93.44 | 208.46 ± 90.16 | 0.952 |  | 204.96 ± 91.22 | 208.42 ± 90.1 | 0.376 |
| 9030 | Urea nitrogen [Mass/volume] in Serum, Plasma or Blood | 15.34 ± 8.86 | 17.16 ± 8.83 | < 0.001 |  | 16.11 ± 8.95 | 17.1 ± 8.8 | 0.011 |

Characteristics ID defines baseline characteristics based on ICD-10 for diagnoses, RxNorm for medications, and CPT for procedures. Oral hypoglycemic agents oral (HS502) Includes Sulfonylureas, alpha-glucosidase inhibitors, Dipeptidyl Peptidase-4 (DPP-4) Inhibitors, and Thiazolidinediones among others. Other Hypoglycemics (HS509) includes GLP1-RAs such as semaglutide, liraglutide, dulaglutide, albiglutide among others.

**Supplementary Table 6.** Full, Before & After Propensity Score Matched Baseline Characteristics for T2DM Patients with NASH Cirrhosis

| T2DM NASH Cirrhosis | | | | | | | | |
| --- | --- | --- | --- | --- | --- | --- | --- | --- |
|  |  | Before Propensity Score Matching | | |  | After Propensity Score Matching | | |
| Characteristic ID | Characteristic Name | Metformin | Metformin + SGLT2-I | P |  | Metformin | Metformin + SGLT2-I | P |
| AI | Age at Index | 57.75 ± 12.58 | 57.99 ± 11.36 | 0.679 |  | 57.84 ± 11.33 | 58.02 ± 11.32 | 0.802 |
| Age | Current Age | 60.92 ± 12.69 | 60.1 ± 11.36 | 0.163 |  | 59.92 ± 11.45 | 60.14 ± 11.31 | 0.755 |
| M | Male | 913 (32.38) | 203 (37.73) | 0.016 |  | 198 (37.01) | 201 (37.57) | 0.850 |
| F | Female | 1722 (61.06) | 313 (58.18) | 0.209 |  | 309 (57.76) | 312 (58.32) | 0.853 |
| UN | Unknown Gender | 185 (6.56) | 22 (4.09) | 0.029 |  | 28 (5.23) | 22 (4.11) | 0.385 |
| 2106-3 | White | 2243 (79.54) | 422 (78.44) | 0.563 |  | 418 (78.13) | 421 (78.69) | 0.824 |
| 1002-5 | American Indian or Alaska Native | 18 (0.64) | 10 (1.86) | < 0.01 |  | 10 (1.87) | 10 (1.87) | 1.000 |
| 2028-9 | Asian | 60 (2.13) | 10 (1.86) | 0.689 |  | 10 (1.87) | 10 (1.87) | 1.000 |
| 2054-5 | Black or African American | 165 (5.85) | 43 (7.99) | 0.059 |  | 45 (8.41) | 42 (7.85) | 0.737 |
| 2076-8 | Native Hawaiian or Other Pacific Islander | 10 (0.36) | 0 (0) | 0.167 |  | 0 (0) | 0 (0) | NA |
| 2131-1 | Unknown Race | 330 (11.7) | 59 (10.97) | 0.625 |  | 60 (11.21) | 59 (11.03) | 0.923 |
| 2135-2 | Hispanic or Latino | 327 (11.6) | 59 (10.97) | 0.675 |  | 55 (10.28) | 59 (11.03) | 0.692 |
| 2186-5 | Not Hispanic or Latino | 1939 (68.76) | 382 (71) | 0.302 |  | 385 (71.96) | 380 (71.03) | 0.735 |
| UN | Unknown Ethnicity | 554 (19.65) | 97 (18.03) | 0.385 |  | 95 (17.76) | 96 (17.94) | 0.936 |
| N17-N19 | Acute kidney failure and chronic kidney disease | 354 (12.55) | 99 (18.4) | < 0.001 |  | 83 (15.51) | 97 (18.13) | 0.253 |
| F50-F59 | Behavioral syndromes associated with physiological disturbances and physical factors | 263 (9.33) | 50 (9.29) | 0.981 |  | 50 (9.35) | 50 (9.35) | 1.000 |
| K00-K95 | Diseases of the digestive system | 2634 (93.4) | 522 (97.03) | < 0.01 |  | 523 (97.76) | 519 (97.01) | 0.444 |
| E00-E89 | Endocrine, nutritional and metabolic diseases | 2667 (94.57) | 521 (96.84) | 0.028 |  | 513 (95.89) | 518 (96.82) | 0.415 |
| I50 | Heart failure | 249 (8.83) | 76 (14.13) | < 0.001 |  | 72 (13.46) | 75 (14.02) | 0.790 |
| I10-I16 | Hypertensive diseases | 2131 (75.57) | 442 (82.16) | < 0.001 |  | 424 (79.25) | 439 (82.06) | 0.246 |
| I20-I25 | Ischemic heart diseases | 599 (21.24) | 148 (27.51) | < 0.01 |  | 140 (26.17) | 146 (27.29) | 0.679 |
| F10-F19 | Mental and behavioral disorders due to psychoactive substance use | 536 (19.01) | 128 (23.79) | 0.011 |  | 120 (22.43) | 126 (23.55) | 0.663 |
| E70-E88 | Metabolic disorders | 2223 (78.83) | 464 (86.25) | < 0.001 |  | 456 (85.23) | 461 (86.17) | 0.662 |
| E13 | Other specified diabetes mellitus | 126 (4.47) | 33 (6.13) | 0.095 |  | 32 (5.98) | 33 (6.17) | 0.898 |
| E65-E68 | Overweight, obesity and other hyperalimentation | 1819 (64.5) | 389 (72.3) | < 0.001 |  | 370 (69.16) | 386 (72.15) | 0.283 |
| 1003143 | Surgery | 2259 (80.11) | 462 (85.87) | < 0.01 |  | 443 (82.8) | 459 (85.79) | 0.179 |
| 1006964 | Surgical Procedures on the Digestive System | 1282 (45.46) | 293 (54.46) | < 0.001 |  | 283 (52.9) | 291 (54.39) | 0.624 |
| CV800 | Ace inhibitors | 1184 (41.99) | 274 (50.93) | < 0.001 |  | 273 (51.03) | 271 (50.65) | 0.903 |
| CV805 | Angiotensin ii inhibitor | 807 (28.62) | 187 (34.76) | < 0.01 |  | 185 (34.58) | 185 (34.58) | 1.000 |
| CV300 | Antiarrhythmics | 1425 (50.53) | 321 (59.66) | < 0.001 |  | 314 (58.69) | 319 (59.63) | 0.756 |
| BL110 | Anticoagulants | 972 (34.47) | 225 (41.82) | < 0.01 |  | 209 (39.06) | 223 (41.68) | 0.383 |
| CV490 | Antihypertensives,other | 508 (18.01) | 123 (22.86) | < 0.01 |  | 111 (20.75) | 122 (22.8) | 0.415 |
| HS503 | Antihypoglycemics | 727 (25.78) | 191 (35.5) | < 0.001 |  | 163 (30.47) | 189 (35.33) | 0.091 |
| CV350 | Antilipemic agents | 1821 (64.57) | 420 (78.07) | < 0.001 |  | 426 (79.63) | 417 (77.94) | 0.501 |
| CV100 | Beta blockers/related | 1242 (44.04) | 277 (51.49) | < 0.01 |  | 278 (51.96) | 275 (51.4) | 0.854 |
| CV200 | Calcium channel blockers | 754 (26.74) | 179 (33.27) | < 0.01 |  | 155 (28.97) | 177 (33.08) | 0.146 |
| CV700 | Diuretics | 1338 (47.45) | 295 (54.83) | < 0.01 |  | 271 (50.65) | 292 (54.58) | 0.199 |
| HS509 | Hypoglycemic agents,other | 526 (18.65) | 220 (40.89) | < 0.001 |  | 221 (41.31) | 217 (40.56) | 0.804 |
| HS501 | Insulin | 1165 (41.31) | 309 (57.44) | < 0.001 |  | 283 (52.9) | 306 (57.2) | 0.158 |
| HS502 | Oral hypoglycemic agents,oral | 2352 (83.4) | 514 (95.54) | < 0.001 |  | 512 (95.7) | 511 (95.51) | 0.881 |
| BL117 | Platelet aggregation inhibitors | 1194 (42.34) | 283 (52.6) | < 0.001 |  | 267 (49.91) | 281 (52.52) | 0.392 |
| 9044 | Alanine aminotransferase [Enzymatic activity/volume] in Serum, Plasma or Blood | 59.98 ± 52.38 | 53.06 ± 45.16 | < 0.01 |  | 56.12 ± 46.51 | 53.13 ± 45.29 | 0.337 |
| 9045 | Albumin [Mass/volume] in Serum, Plasma or Blood | 4.11 ± 0.49 | 4.17 ± 0.42 | 0.012 |  | 4.13 ± 0.49 | 4.17 ± 0.42 | 0.213 |
| 9046 | Alkaline phosphatase [Enzymatic activity/volume] in Serum, Plasma or Blood | 97.8 ± 55.22 | 92.94 ± 39.96 | 0.081 |  | 95.55 ± 48.9 | 93.11 ± 40.01 | 0.423 |
| 16362-6 | Ammonia [Moles/volume] in Plasma | 610.44 ± 3994.4 | 36.81 ± 24 | 0.570 |  | 51.58 ± 40 | 36.81 ± 24 | 0.234 |
| 9047 | Aspartate aminotransferase [Enzymatic activity/volume] in Serum or Plasma | 50.63 ± 41.26 | 44.13 ± 28.13 | < 0.01 |  | 47.96 ± 36.73 | 44.18 ± 28.2 | 0.090 |
| 9048 | Bilirubin.direct [Mass/volume] in Serum or Plasma | 0.21 ± 0.29 | 0.22 ± 0.36 | 0.637 |  | 0.2 ± 0.22 | 0.22 ± 0.36 | 0.524 |
| 9049 | Bilirubin.indirect [Mass/volume] in Serum or Plasma | 0.54 ± 0.56 | 0.55 ± 0.29 | 0.975 |  | 0.5 ± 0.24 | 0.55 ± 0.29 | 0.330 |
| 9050 | Bilirubin.total [Mass/volume] in Serum, Plasma or Blood | 0.65 ± 0.57 | 0.61 ± 0.37 | 0.154 |  | 0.61 ± 0.38 | 0.61 ± 0.37 | 0.922 |
| 9083 | BMI | 35.6 ± 6.76 | 35.83 ± 6.67 | 0.593 |  | 35.63 ± 7.23 | 35.85 ± 6.69 | 0.702 |
| 9081 | Body weight | 223.69 ± 58.81 | 223.65 ± 59.69 | 0.989 |  | 225.82 ± 60.65 | 224.04 ± 59.44 | 0.678 |
| 9022 | Calcium [Mass/volume] in Serum, Plasma or Blood | 9.44 ± 0.63 | 9.51 ± 0.56 | 0.026 |  | 9.46 ± 0.53 | 9.51 ± 0.56 | 0.164 |
| 9024 | Creatinine [Mass/volume] in Serum, Plasma or Blood | 0.91 ± 3.89 | 0.87 ± 0.27 | 0.846 |  | 0.84 ± 0.29 | 0.87 ± 0.27 | 0.120 |
| 9051 | Gamma glutamyl transferase [Enzymatic activity/volume] in Serum or Plasma | 114.94 ± 165.1 | 102.44 ± 138.39 | 0.423 |  | 118.33 ± 208.41 | 103.01 ± 139.36 | 0.487 |
| 8001 | Glomerular filtration rate/1.73 sq M.predicted [Volume Rate/Area] in Serum, Plasma or Blood by Creatinine-based formula (MDRD) | 84.6 ± 24.04 | 83.04 ± 24.03 | 0.210 |  | 84.4 ± 22.95 | 83.14 ± 24.05 | 0.422 |
| 9025 | Glucose [Mass/volume] in Serum, Plasma or Blood | 154.48 ± 66.86 | 173.57 ± 72.65 | < 0.001 |  | 157.97 ± 64.88 | 173.99 ± 72.71 | < 0.001 |
| 9037 | Hemoglobin A1c/Hemoglobin.total in Blood | 7.36 ± 1.7 | 8.07 ± 1.83 | < 0.001 |  | 7.61 ± 1.71 | 8.06 ± 1.82 | < 0.001 |
| 9032 | INR in Plasma or Blood | 1.16 ± 1.91 | 1.09 ± 0.26 | 0.497 |  | 1.35 ± 3.98 | 1.09 ± 0.26 | 0.210 |
| 9020 | Platelets [#/volume] in Blood | 214.52 ± 80.9 | 215.49 ± 83.83 | 0.819 |  | 216.02 ± 80.7 | 214.27 ± 78.11 | 0.745 |
| 9030 | Urea nitrogen [Mass/volume] in Serum, Plasma or Blood | < 0.01 | 13.82 ± 7.01 | 14.95 ± 6.76 |  | 14.15 ± 6.7 | 14.96 ± 6.78 | 0.079 |

Characteristics ID defines baseline characteristics based on ICD-10 for diagnoses, RxNorm for medications, and CPT for procedures. Oral hypoglycemic agents oral (HS502) Includes Sulfonylureas, alpha-glucosidase inhibitors, Dipeptidyl Peptidase-4 (DPP-4) Inhibitors, and Thiazolidinediones among others. Other Hypoglycemics (HS509) includes GLP1-RAs such as semaglutide, liraglutide, dulaglutide, albiglutide among others.

**Supplemental Table 7.** Cirrhosis Cohort Etiology

|  |  | Before Propensity Score Matching | | |  | After Propensity Score Matching | | |
| --- | --- | --- | --- | --- | --- | --- | --- | --- |
| Code | Cirrhosis Etiology | Metformin | Metformin + SGLT2i | P |  | Metformin | Metformin + SGLT2i | P |
| K76.0 | Fatty (change of) liver, not elsewhere classified | 6004 (28.22) | 614 (43.57) | < 0.001 |  | 566 (40.06) | 575 (40.69) | 0.730 |
| K75.8 | Other specified inflammatory liver diseases | 2251 (10.03) | 321 (22.8) | < 0.001 |  | 306 (21.66) | 274 (19.39) | 0.136 |
| K70.3 | Alcoholic cirrhosis of liver | 1148 (5.1) | 106 (7.54) | < 0.001 |  | 99 (7.08) | 96 (6.86) | 0.8246216 |
| B18.2 | Chronic viral hepatitis C | 3005 (13.35) | 196 (13.88) | 0.533 |  | 179 (12.67) | 188 (13.3) | 0.615 |
| B18.1 | Chronic viral hepatitis B without delta-agent | 23 (0.11) | 0 (0) | 0.179 |  | 0 (0) | 0 (0) | - |
| B18.0 | Chronic viral hepatitis B with delta-agent | 495 (2.22) | 36 (2.15) | 0.854 |  | 28 (1.98) | 30 (2.12) | 0.791 |
| K74.6 | Other and unspecified cirrhosis of liver | 5617 (24.95) | 790 (56.31) | < 0.001 |  | 689 (48.76) | 686 (48.55) | 0.910 |
| K75.4 | Autoimmune hepatitis | 225 (1) | 22 (1.32) | 0.218 |  | 24 (1.7) | 20 (1.42) | 0.543 |
| E83.11 | Hemochromatosis | 209 (0.93) | 19 (1.14) | 0.401 |  | 15 (1.06) | 14 (0.99) | 0.852 |
| E88.01 | Alpha-1-antitrypsin deficiency | 39 (0.18) | 10 (0.6) | < 0.001 |  | 10 (0.71) | 10 (0.71) | 1 |

Baseline diagnoses of T2DM (E11) patients with cirrhosis (K70.3, K74, K74.6, K74.2) on metformin (n=22,515) or metformin and SGLT2-I (n=1,411) before and after propensity score matching. NASH cirrhosis is defined by patients with both ICD-10 codes K76.0 and K75.8 concurrently. Values are n (%).

**Supplemental Table 8.** Before & After Propensity Score Matching, Mortality Outcomes

| Kaplan Meier Estimates | | | | | | | | | |
| --- | --- | --- | --- | --- | --- | --- | --- | --- | --- |
| **5-Year Survival Rate (%)** | Before Propensity Score Matching | | | |  | After Propensity Score Matching | | | |
|  | Metformin | Metformin + SGLT2-I | P | HR (95CI) |  | Metformin | Metformin + SGLT2-I | P | HR (95%CI) |
| All | 84.6 | 90.39 | <0.001 | 0.59 (0.44-0.78) |  | 82.09 | 90.38 | 0.002 | 0.57 (0.41-0.81) |
| Subgroup |  |  |  | - |  |  |  |  | - |
| Men | 79.69 | 85.12 | 0.004 | 0.6 (0.42-0.85) |  | 77.76 | 85.09 | 0.031 | 0.62 (0.4-0.96) |
| Women | 88.05 | 94.83 | 0.012 | 0.55 (0.35-0.88) |  | 84.52 | 94.8 | < 0.01 | 0.46 (0.26-0.79) |
| White | 83.74 | 93.98 | <0.001 | 0.49 (0.34-0.69) |  | 80.08 | 93.96 | < 0.001 | 0.39 (0.26-0.6) |
| Non-White | 89.74 | 82.72 | 0.438 | 0.74 (0.35-1.59) |  | 83.22 | 82.58 | 0.468 | 0.7 (0.27-1.85) |
| Hispanic | 87.39 | 98.36 | 0.05 | 0.27 (0.07-1.11) |  | 96.4 | 98.18 | 0.464 | 0.53 (0.1-2.94) |
| Non-Hispanic | 84.61 | 93.84 | <0.001 | 0.5 (0.35-0.72) |  | 75.58 | 93.83 | < 0.001 | 0.40 (0.27-0.62) |
| Age 39-59 | 91.28 | 94.08 | 0.505 | 0.83 (0.47-1.45) |  | 92.73 | 94.26 | 0.305 | 0.68 (0.32-1.43) |
| Age 60-80 | 83.03 | 88.63 | 0.003 | 0.6 (0.42-0.84) |  | 82.66 | 88.62 | 0.011 | 0.58 (0.38-0.88) |
| NASH | 93.44 | 89.89 | 0.071 | 0.44 (0.18-1.1) |  | 93.82 | 89.89 | 0.746 | 0.82 (0.25-2.7) |

K-M probabilities values are percent free of death. P values indicate P Log-rank Test. ^†^T2DM Cirrhosis patients treated with Metformin and SGLT2-I were further divided into demographic subgroups and were propensity score matched to the Metformin group by sex, race, ethnicity, and age groups. T2DM NASH Cirrhosis patients treated with Metformin and SGLT2-I were propensity score matched to the NASH Cirrhosis Metformin group. Abbreviations. T2DM, Type 2 Diabetes Mellitus; Sodium glucose cotransporter-2 inhibitors; NASH, Non-Alcoholic Steatohepatitis; HR, Hazard Ratio; CI, Confidence Interval; K-M, Kaplan Meier

**Supplemental Table 9.** Before & After Propensity Score Matching, Composite Hepatic Decompensation Outcomes

| Kaplan Meier Estimates | | | | | | | | | |
| --- | --- | --- | --- | --- | --- | --- | --- | --- | --- |
| **5-Year Absence of Hepatic Decompensation (%) ^§^** | Before Propensity Score Matching | | | |  | After Propensity Score Matching | | | |
|  | Metformin | Metformin + SGLT2-I | P | HR (95%CI) |  | Metformin | Metformin + SGLT2-I | P | HR (95%CI) |
| All | 88.78 | 92.24 | 0.115 | 0.63 (0.43-0.93) |  | 87.62 | 92.22 | 0.017 | 0.63 (0.43-0.93) |
| Subgroup^†^ |  |  |  | - |  |  |  |  | - |
| Men | 86.79 | 87.81 | 0.607 | 0.83 (0.51-1.37) |  | 87.42 | 87.78 | 0.477 | 0.83 (0.51-1.37) |
| Women | 90.27 | 96.05 | 0.045 | 0.57 (0.29-1.09) |  | 88.72 | 96.02 | 0.086 | 0.57 (0.29-1.09) |
| White | 88.2 | 92.53 | 0.053 | 0.49 (0.31-0.76) |  | 87.43 | 92.51 | < 0.01 | 0.49 (0.31-0.76) |
| Non-White | 90.04 | 94.49 | 0.219 | 0.43 (0.15-1.23) |  | 93.05 | 94.31 | 0.105 | 0.43 (0.15-1.23) |
| Hispanic | 89.42 | 98.55 | 0.177 | 0.39 (0.08-1.92) |  | 91.82 | 98.4 | 0.230 | 0.39 (0.08-1.92) |
| Non-Hispanic | 89.24 | 91.54 | 0.171 | 0.73 (0.46-1.16) |  | 87.84 | 91.53 | 0.185 | 0.73 (0.46-1.16) |
| Age 39-59 | 91.87 | 95.16 | 0.538 | 1.18 (0.51-2.7) |  | 94.86 | 95.08 | 0.708 | 1.18 (0.51-2.7) |
| Age 60-80 | 87.21 | 90.99 | 0.105 | 0.53 (0.33-0.85) |  | 85.84 | 90.98 | < 0.01 | 0.53 (0.33-0.85) |
| NASH | 93.08 | 97.43 | 0.11 | 0.52 (0.22-1.22) |  | 95.39 | 97.43 | 0.124 | 0.52 (0.22-1.22) |

K-M probabilities values are percent free of decompensation. P values indicate P Log-rank Test. T2DM Cirrhosis patients treated with Metformin and SGLT2-I were further divided into demographic subgroups and were propensity score matched to the Metformin group by sex, race, ethnicity, and age groups. T2DM NASH Cirrhosis patients treated with Metformin and SGLT2-I were propensity score matched to the NASH Cirrhosis Metformin group. Abbreviations. Composite Hepatic Decompensation is defined as any instance of ascites, variceal bleeding, or hepatic encephalopathy. T2DM, Type 2 Diabetes Mellitus; Sodium glucose cotransporter-2 inhibitors; NASH, Non-Alcoholic Steatohepatitis; HR, Hazard Ratio; CI, Confidence Interval; K-M, Kaplan Meier

**Supplemental Table 10.** Before & After Propensity Score Matching, Hepatocellular Carcinoma outcomes

| Kaplan Meier Estimates | | | | | | | | | |
| --- | --- | --- | --- | --- | --- | --- | --- | --- | --- |
| **5-Year Absence of Hepatocellular Carcinoma (%)** | Before Propensity Score Matching | | | |  | After Propensity Score Matching | | | |
|  | Metformin | Metformin + SGLT2-I | P | HR (95%CI) |  | Metformin | Metformin + SGLT2-I | P | HR (95%CI) |
| All | 92.86 | 98.62 | 0.009 | 0.46 (0.25-0.84) |  | 93.75 | 98.61 | 0.017 | 0.43 (0.21-0.88) |
| Subgroup |  |  |  | - |  |  |  |  | - |
| Men | 94.69 | 99.64 | 0.011 | 0.2 (0.05-0.79) |  | 86.91 | 99.64 | 0.051 | 0.24 (0.05-1.14) |
| Women | 91.2 | 97.74 | 0.177 | 0.64 (0.33-1.23) |  | 85.02 | 97.72 | 0.106 | 0.52 (0.23-1.16) |
| White | 91.97 | 98.19 | 0.064 | 0.56 (0.3-1.04) |  | 92.71 | 98.18 | 0.029 | 0.44 (0.21-0.93) |
| Non-White | 94.96 | 99.53 | 0.177 | 0.28 (0.04-2) |  | 96.87 | 99.49 | 0.100 | 0.2 (0.02-1.69) |
| Hispanic | 93.57 | 99.25 | 0.32 | 0.38 (0.05-2.78) |  | 95.54 | 99.17 | 0.385 | 0.38 (0.04-3.7) |
| Non-Hispanic | 91.55 | 98.19 | 0.03 | 0.51 (0.27-0.94) |  | 93.32 | 98.18 | 0.037 | 0.46 (0.22-0.97) |
| Age 39-59 | 90.6 | 99.45 | 0.013 | 0.2 (0.05-0.83) |  | 95.21 | 99.44 | 0.134 | 0.31 (0.06-1.56) |
| Age 60-80 | 95.33 | 98.64 | 0.155 | 0.56 (0.25-1.27) |  | 97.18 | 98.64 | 0.051 | 0.4 (0.16-1.03) |
| NASH | 95.63 | 98.72 | 0.016 | 0.13 (0.02-0.93) |  | 93.61 | 98.72 | 0.120 | 0.08 (0.01-0.62) |

K-M probabilities values are percent free of hepatocellular carcinoma. P values indicate P Log-rank Test. T2DM Cirrhosis patients treated with Metformin and SGLT2-I were further divided into demographic subgroups and were propensity score matched to the Metformin group by sex, race, ethnicity, and age groups. T2DM NASH Cirrhosis patients treated with Metformin and SGLT2-I were propensity score matched to the NASH Cirrhosis Metformin group. Abbreviations. T2DM, Type 2 Diabetes Mellitus; Sodium glucose cotransporter-2 inhibitors; NASH, Non-Alcoholic Steatohepatitis; HR, Hazard Ratio; CI, Confidence Interval; K-M, Kaplan Meier

**Supplemental Table 11.** 6 months, 1 year, 2 year, 3 year, and 5 year Kaplan Meier Estimates for T2DM Patients with Cirrhosis

| **Cumulative Outcome Probabilities for T2DM Patients With Cirrhosis** | | | | | | | | | |
| --- | --- | --- | --- | --- | --- | --- | --- | --- | --- |
|  | **Before Propensity Score Matching** | | | |  | **After Propensity Score Matching** | | | |
|  | **Metformin** | **Metformin + SGLT2-I** | **P** | **HR (95%CI)** |  | **Metformin** | **Metformin + SGLT2-I** | **P** | **HR (95%CI)** |
| **Outcome** | **(n = 22,515)** | **(n = 1,411)** |  |  |  | **(n = 1,403)** | **(n = 1,403)** |  |  |
| **All-Cause Mortality** |  |  |  |  |  |  |  |  |  |
| 6 Months | 95.35 | 97.36 | < 0.01 | 0.53 (0.37-0.78) |  | 94.84 | 97.35 | < 0.01 | 0.47 (0.3-0.74) |
| 1 year | 93.58 | 96.44 | < 0.01 | 0.54 (0.39-0.75) |  | 93.45 | 96.43 | < 0.01 | 0.51 (0.34-0.76) |
| 2 years | 90.93 | 93.71 | < 0.01 | 0.6 (0.45-0.81) |  | 90.61 | 93.70 | 0.002 | 0.57 (0.4-0.81) |
| 3 years | 88.67 | 92.83 | < 0.01 | 0.6 (0.45-0.79) |  | 89.67 | 92.82 | 0.002 | 0.58 (0.41-0.83) |
| 5 years | 84.60 | 90.39 | <0.001 | 0.59 (0.44-0.78) |  | 82.09 | 90.38 | 0.002 | 0.57 (0.41-0.81) |
| **Hepatic Decompensation** |  |  |  | - |  |  |  |  | - |
| 6 Months | 96.75 | 97.96 | 0.032 | 0.63 (0.41-0.96) |  | 96.41 | 97.95 | 0.028 | 0.56 (0.33-0.94) |
| 1 year | 95.50 | 96.79 | 0.052 | 0.7 (0.48-1.01) |  | 94.48 | 96.77 | 0.016 | 0.58 (0.37-0.91) |
| 2 years | 93.61 | 93.58 | 0.264 | 0.84 (0.62-1.14) |  | 91.35 | 93.56 | 0.045 | 0.67 (0.45-0.99) |
| 3 years | 91.73 | 93.58 | 0.123 | 0.79 (0.57-1.06) |  | 88.81 | 93.56 | 0.015 | 0.62 (0.42-0.92) |
| 5 years | 88.78 | 92.24 | 0.115 | 0.78 (0.58-1.06) |  | 87.62 | 92.22 | 0.017 | 0.63 (0.43-0.93) |
| **Hepatocellular Carcinoma** |  |  |  | - |  |  |  |  | - |
| 6 Months | 99.08 | 99.19 | 0.833 | 0.93 (0.48-1.82) |  | 98.78 | 99.19 | 0.516 | 0.75 (0.32-1.79) |
| 1 year | 98.41 | 99.19 | 0.182 | 0.64 (0.33-1.23) |  | 98.08 | 99.19 | 0.121 | 0.53 (0.24-1.19) |
| 2 years | 97.08 | 98.62 | 0.064 | 0.57 (0.31-1.04) |  | 97.33 | 98.61 | 0.113 | 0.56 (0.27-1.16) |
| 3 years | 95.13 | 98.62 | 0.015 | 0.49 (0.27-0.88) |  | 94.95 | 98.61 | 0.022 | 0.45 (0.22-0.91) |
| 5 years | 92.86 | 98.62 | 0.009 | 0.46 (0.25-0.84) |  | 93.75 | 98.61 | 0.017 | 0.43 (0.21-0.88) |

K-M probabilities values are percent free of death, decompensation, and hepatocellular carcinoma. P values indicate P Log-rank Test. HR, Hazard Ratio; CI, Confidence Interval.

**Supplemental Table 12.** 6 months, 1 year, 2 year, 3 year, and 5 year Kaplan Meier Estimates for T2DM Patients with NASH Cirrhosis

| **Cumulative Outcome Probabilities for T2DM Patients with NASH Cirrhosis** | | | | | | | | | |
| --- | --- | --- | --- | --- | --- | --- | --- | --- | --- |
|  | **Before Propensity Score Matching** | | | |  | **After Propensity Score Matching** | | | |
|  | **Metformin** | **Metformin + SGLT2-I** | **P** | **HR (95%CI)** |  | **Metformin** | **Metformin + SGLT2-I** | **P** | **HR (95%CI)** |
| **Outcome** | **(n = 2,820)** | **(n = 538)** |  |  |  | **(n = 535)** | **(n = 535)** |  |  |
| **All-Cause Mortality** |  |  |  |  |  |  |  |  |  |
| 6 Months | 99.09 | 99.80 | 0.101 | 0.22 (0.03-1.61) |  | 99.41 | 99.80 | 0.301 | 0.32 (0.03-3.12) |
| 1 year | 98.75 | 99.33 | 0.243 | 0.5 (0.15-1.64) |  | 99.41 | 99.33 | 0.957 | 0.95 (0.19-4.76) |
| 2 years | 97.5 | 98.88 | 0.103 | 0.44 (0.16-1.22) |  | 99.03 | 98.88 | 0.952 | 0.96 (0.24-3.85) |
| 3 years | 96.73 | 98.88 | 0.059 | 0.39 (0.14-1.08) |  | 98.28 | 98.88 | 0.692 | 0.77 (0.21-2.86) |
| 5 years | 93.44 | 89.89 | 0.071 | 0.44 (0.18-1.1) |  | 93.82 | 89.89 | 0.746 | 0.82 (0.25-2.7) |
| **Hepatic Decompensation** |  |  |  | - |  |  |  |  | - |
| 6 Months | 98.69 | 99.38 | 0.185 | 0.46 (0.14-1.49) |  | 97.96 | 99.38 | 0.046 | 0.29 (0.08-1.06) |
| 1 year | 98.25 | 99.15 | 0.152 | 0.48 (0.17-1.33) |  | 97.68 | 99.15 | 0.062 | 0.35 (0.11-1.11) |
| 2 years | 96.70 | 97.43 | 0.269 | 0.66 (0.32-1.37) |  | 96.88 | 97.43 | 0.245 | 0.6 (0.25-1.43) |
| 3 years | 95.71 | 97.43 | 0.16 | 0.6 (0.29-1.23) |  | 95.39 | 97.43 | 0.124 | 0.52 (0.22-1.22) |
| 5 years | 93.08 | 97.43 | 0.11 | 0.56 (0.27-1.15) |  | 95.39 | 97.43 | 0.124 | 0.52 (0.22-1.22) |
| **Hepatocellular Carcinoma** |  |  |  | - |  |  |  |  | - |
| 6 Months | 99.42 | 100 | 0.092 | - |  | 98.91 | 100 | 0.022 | - |
| 1 year | 99.01 | 100 | 0.037 | - |  | 98.02 | 100 | 0.004 | - |
| 2 years | 98.42 | 100 | 0.016 | - |  | 97.6 | 100 | 0.002 | - |
| 3 years | 97.21 | 100 | 0.007 | - |  | 96.21 | 100 | 0.001 | - |
| 5 years | 95.63 | 98.72 | 0.016 | 0.13 (0.02-0.93) |  | 93.61 | 98.72 | 0.120 | 0.08 (0.01-0.62) |

K-M probabilities values are percent free of death, decompensation, and hepatocellular carcinoma. P values indicate P Log-rank Test. HR, Hazard Ratio; CI, Confidence Interval.
